# Co-Pyrolysis of Polyolefins and Silicone Rubber: Effects on Mass Balancing, Product Distribution, and Potential Siloxane Recovery

**DOI:** 10.3390/polym18080989

**Published:** 2026-04-18

**Authors:** Lukas Eigenschink, Wolfgang Eder, Matthias Mastalir, Michael Harasek, Christian Paulik

**Affiliations:** 1 Institute for Chemical Technology of Organic Materials, Johannes Kepler University Linz, Altenberger Straße 69, 4040 Linz, Austria; christian.paulik@jku.at; 2 Institute of Chemical, Environmental and Bioscience Engineering, Vienna University of Technology (TU Wien), Getreidemarkt 9/166, 1060 Vienna, Austria; michael.harasek@tuwien.ac.at; 3 OMV Downstream GmbH, Mannswörther Straße 28, 2320 Schwechat, Austria; wolfgangmathias.eder@omv.com (W.E.); matthias.mastalir@omv.com (M.M.)

**Keywords:** polyolefins, silicone rubber, co-pyrolysis, chemical recycling

## Abstract

Co-pyrolysis of polyolefins (LDPE, PP, PS) mixed with silicone rubber (SR) was investigated using a laboratory-scale pyrolysis apparatus to evaluate product composition, synergistic interactions, and siloxane recovery potential. Synergistic effects were assessed by comparing experimental mass balances and product distributions with calculated values derived from individual polymer pyrolysis. Co-pyrolysis resulted in a reduction in liquid yield and an increase in gaseous products and solid residue compared to calculated values, with liquid yields decreasing by up to ≈15 wt% at high SR content. This shift was accompanied by an enrichment in lighter hydrocarbons in both phases, reaching up to a ≈18% relative increase at high SR content, and by a redistribution towards smaller cyclic siloxanes. Chromatographic analysis confirmed that no new compounds were formed, but the proportion of low molecular weight species increased with silicone content. These effects are attributed to the distinct thermal behavior of the polymers, as silicone rubber does not melt but becomes brittle, allowing molten polyolefins to infiltrate surface cracks and prolong residence time, thereby promoting secondary cracking. Furthermore, recovery of hexamethylcyclotrisiloxane (D_3_), the primary silicone pyrolysis product, was demonstrated from the liquid co-pyrolysis products via solvent-assisted filtration using ethanol, achieving purities above 99.5% and recovery rates up to ≈75% compared to other possible methods. These findings provide insights into co-pyrolysis behavior and offer a basis for developing strategies for the recovery of siloxane and advanced recycling of mixed polymer waste.

## 1. Introduction

The global proliferation of plastic waste presents a significant environmental and technological challenge. Global plastic production exceeded 400 million metric tons in 2022 [1] and is projected to reach approximately 884 million metric tons by 2050 [2]. This anticipated growth will significantly increase the volume of mixed plastic waste, complicating recycling processes due to the diverse chemical compositions and thermal behaviors of different polymers. Conventional mechanical recycling methods are often inefficient or infeasible for mixed or contaminated plastic streams, leading to a growing interest in thermochemical approaches, such as pyrolysis [3]. Pyrolysis offers a promising route for converting plastic waste into valuable chemical feedstocks and fuels, but its effectiveness is highly dependent on the nature of the polymers involved and their interactions during thermal degradation [4].

Among polymers present in post-consumer and industrial waste, polyolefins (PO), primarily polyethylene (LDPE, HDPE, LLDPE), polypropylene (PP), and polystyrene (PS) are the most abundant, owing to their extensive use in packaging, automotive components, and consumer goods [5]. Collectively, they account for approximately 50 wt% of global plastic production [6]. These materials are characterized by high hydrogen-to-carbon ratios and relatively simple hydrocarbon backbones, which make them suitable candidates for pyrolytic conversion into liquid hydrocarbons and gaseous fuels [7,8,9,10]. However, the presence of non-polyolefin materials in mixed waste streams introduces complexity into the pyrolysis process and could alter the product distribution and yield [11,12,13,14].

Silicone rubbers (SRs), particularly polydimethylsiloxane (PDMS)-based, are widely used in medical devices, kitchenware, electronics, sealants, and high-performance coatings due to their thermal stability, flexibility, and chemical inertness [15]. Silicone rubbers are frequently incorporated into polymer formulations as processing aids to improve flow characteristics during extrusion and molding and are also added to enhance flame retardancy and modify combustion behavior due to their thermal stability and inert residue formation [16]. Their incorporation into consumer and industrial products often results in silicone-containing waste being co-disposed with polyolefins. Despite their prevalence, the pyrolytic behavior of silicones, especially in mixed polymer systems, remains underexplored. This gap in knowledge is important, as silicones exhibit distinct degradation pathways compared to hydrocarbon-based polymers [17], potentially influencing the yield and composition of pyrolysis products when processed together.

During co-pyrolysis of mixed polymer streams, interactive effects between components may redirect degradation routes, leading to substantial shifts in the composition and distribution of degradation products. At the same time, co-pyrolysis can be beneficial by reducing the apparent activation energy, thereby lowering the required reaction temperature or enhancing the formation of target products through alternative reaction pathways. These synergistic effects are particularly advantageous, as they enable the direct processing of mixed plastic feeds without the need for complete separation into individual polymer types [11,14,18,19,20]. While co-pyrolysis of different polymers [21,22], waste material [23,24], and additives [25] have been studied, the behavior of silicone–polyolefin mixtures under pyrolytic conditions is not well understood. Investigating these interactions is essential for optimizing thermochemical recycling processes and improving the recovery of valuable compounds from mixed plastic waste.

One of the key degradation products of PDMS-based silicones during pyrolysis is hexamethylcyclotrisiloxane (D_3_), a cyclic trimer formed through backbiting reactions [17]. D_3_ is a commercially valuable compound used as a precursor in the synthesis of silicone elastomers, resins, and surfactants [26,27,28]. Its recovery from waste streams not only adds economic value to the pyrolysis process but also supports circular economy principles by enabling the reuse of silicone-derived materials. However, the formation and recovery of D_3_ in the presence of polyolefins have not been systematically studied, and it remains unclear how co-pyrolysis conditions affect its formation, the possibility of recovery, and yield.

This study aims to address these gaps by investigating the co-pyrolysis behavior of silicone rubber and polyolefins under controlled thermal conditions. Through a series of individual and mixed pyrolysis experiments, we examine the influence of polymer interactions on product distribution, with a particular focus on the effect on mass balancing, gaseous and liquid product distribution, and the recovery of D_3_. By comparing the pyrolysis profiles of pure and blended polymers, we seek to identify potential synergistic effects and assess the feasibility of selective recovery of valuable compounds from mixed plastic waste. The findings contribute to a deeper understanding of mixed polymer pyrolysis and offer insights into the development of more efficient recycling technologies for complex waste streams.

## 2. Materials and Methods

### 2.1. Material

PS (product type: Polystyrene Crystal 1540, M_w_ = 366,847 g mol^−1^, M_n_ = 62,030 g mol^−1^) was purchased from TotalEnergies (Brussels, Belgium). LDPE (M_w_ ~110,000 g mol^−1^, average M_n_ ~35,000 g mol^−1^) and PP (M_w_ ~250,000 g mol^−1^, average M_n_ ~67,000 g mol^−1^) were provided by Sigma Aldrich (St. Lous, MO, USA). The polyolefins were provided additive-free as white pellets. Silicone rubber, ultra-high molecular weight polydimethylsiloxane (UHMW-PDMS) of the product type Genioplast^®^ Pellet S, was provided as opaque pellets containing approx. 70 wt% silicone polymer and 30 wt% fumed silica by Wacker Chemie AG (Munich, Germany). Genioplast^®^ Pellet S was chosen because one of its applications is its use as a processing aid in polyolefins. In addition, this choice reflects industrial practice, as commercially available silicone rubbers typically contain inorganic fillers. Before the pyrolysis experiments and mixing of the polymers, all the polymers were ground mechanically. For polymer mixtures, the ground polymers were physically combined and mixed by repeated stirring. A homogeneous mixture was obtained as the melting of the polyolefin facilitated effective component dispersion. Hexamethylcyclotrisiloxane (D_3_, 98%) was provided by Sigma Aldrich (St. Louis, MO, USA).

### 2.2. Laboratory Pyrolysis

The pyrolysis was conducted in a static N_2_ atmosphere using a laboratory-scale batch reactor implemented using standard glassware. A total of 10 g of the pure polymers and PO:SR mixtures with three varying mass ratios were used. To ensure reproducibility, the pyrolysis of each pure polymer was conducted in three independent experimental runs. The polymer mixtures were placed stationary in a round-bottom flask (flask 1) and heated using a high-power external thermal source applied directly to the wall of flask 1. The reactor temperature was measured at the outer surface of the flask, using an infrared pyrometer. The effective heating rate was calculated to be approximately 40 °C s^−1^. This heating rate is comparable to industrial reactors, classified in the range of intermediate to fast pyrolysis [29,30]. The reactor was heated to a maximum temperature of 600 °C and maintained at this temperature for 10 min, after which no further gas evolution was observed. The residue fraction remained in flask 1, whereas the evolved gas was cooled through a distillation condenser (20 °C), leading to solid, liquid, and wax products being collected in another round-bottom flask (flask 2). The gaseous products, which are non-condensable at 20 °C, were collected in a gas sample bag. For the mass balancing, the yield of the gaseous fraction was calculated through the difference between the mass of the sample and the liquid/solid/wax products, together with the residue. The arithmetic mean of individual polymer yield profiles served as the basis for calculating an expected mass balance for co-pyrolyzed mixtures, enabling comparative analysis. A final pyrolysis temperature of 600 °C was selected because the primary decomposition of LDPE, PP [31], and PS [32] typically occurs in the range of 400–480 °C, whereas the thermal degradation of silicone rubber extends over a broader interval of approximately 400–600 °C [33], with a peak decomposition temperature near 550 °C. The peak degradation temperatures (Table S1) and degradation intervals of the used polymers were tested by TGA, which is depicted in Figures S1–S5. Choosing 600 °C in combination with a high heating rate ensures substantial overlap between the degradation regimes of the polyolefins and the silicone rubber, thereby enabling a more precise investigation of potential synergistic effects. In contrast, significantly slower heating rates could reduce the extent of such interactions and thus diminish observable synergy effects.

### 2.3. Isolation of Hexamethylcyclotrisiloxane (D_3_)

The liquid co-pyrolysis products obtained from all experiments (mass ratio 1:1) were subjected to vacuum distillation. Under the applied conditions (200 mbar, 50 °C), the D_3_-rich fractions were isolated predominantly as colorless crystalline solids accompanied by small amounts of colorless liquid. The distillates were subsequently cooled to 4 °C for 1 h and decanted. The resulting crystalline sample was then purified either by washing at 4 °C or by recrystallization, which involved dissolution at the boiling point of the respective solvent followed by cooling to 4 °C for 1 h. For solvent-assisted filtration, the sample was filtered using a 0.2 µm PTFE syringe filter with precooled solvent (4 °C). All purified materials, obtained via washing, recrystallization, or solvent-assisted filtration, were finally dried under vacuum (4 mbar, 10 min). To assess the recovery of D_3_, distillation fractions from the pure polyolefin pyrolysis products were obtained at 200 mbar and 55 °C and mixed with varying amounts of D_3_. The samples were heated to 70 °C to ensure complete dissolution of D_3_, after which they were processed using the same procedures applied to distillation fractions obtained from PO:SR pyrolysis.

### 2.4. Gas Chromatography–Mass Spectrometry (GC-MS)

The obtained liquid products were diluted with dichloromethane and subsequently analyzed by GC-MS. The products were separated using a gas chromatograph (Thermo Finnigan Trace GC, Thermo Fisher Scientific, San Jose, CA, USA) equipped with a capillary column (Rtx-5MS 5% diphenyl, 95% dimethylpolysiloxane, 30 m × 0.25 mm inner diameter × 0.25 µm film thickness) using a 1.5 mL min^−1^ He flow. Entering the inlet (280 °C, split flow 50 mL min^−1^, split ratio 33), the sample was separated using a temperature program starting at 40 °C and held for 1 min, which was followed by a heating rate of 15 °C min^−1^ to 300 °C, which was held for 10 min. The mass spectra were recorded using a PolarisQ iontrap mass spectrometer (Thermo Finnigan) under 70 eV electron ionization in a *m*/*z* range of 50–800 at 1.96 scans sec^−1^. The compounds detected through GC-MS were identified using the NIST library, reference substances, and literature data.

### 2.5. Gas Chromatography–Flame Ionization Detector–Thermal Conductivity Detector (GC-FID-TCD)

The gaseous products were analyzed according to OENORM15984 (2022) [34] with a Fast RGA—Refinery Gas Analyzer (Agilent 7890A, Santa Clara, CA, USA) for the detection of hydrocarbons, CO, CO_2_, N_2_, O_2_, and H_2_ equipped with a capillary column (HayeSep Q 35 m × 320 µm × 8 µm). Entering the inlet (200 °C, split flow 767 mL min^−1^, split ratio 150), the sample was separated using a temperature program starting at 80 °C and was held for 2 min, followed by a heating rate of 35 °C min^−1^ to 185 °C, which was held for 2 min. The compounds were quantified using a thermal conductivity detector (TCD) and flame ionization detector (FID) using reference substances.

### 2.6. Nuclear Magnetic Resonance Spectroscopy (NMR)

To identify silicon compounds formed during pyrolysis, in addition to GC-MS, NMR spectroscopy was performed. NMR spectra were recorded using a Bruker Avance 400 (400 MHz) in CDCl_3_. CDCl_3_ was stored over silver foil and filtered through a short plug of basic alumina just before use. All chemical shifts are reported in ppm and were referenced externally to Si(CH_3_)_4_ for ^29^Si-NMR.

## 3. Results and Discussion

This study investigates the pyrolysis behavior of pure polymers and their mixtures, with emphasis on mass balance, product composition, and phase distribution. Additionally, methods for the isolation of D_3_ are also discussed. In order to yield PO and SR pyrolysis products and to study potential synergistic effects, the pure polymers and polymer blends were pyrolyzed on a laboratory scale. The liquid and gaseous pyrolysis products were characterized, and through the pure polymer pyrolysis data, the mass balancing and product distribution were calculated and compared to the experimental data.

### 3.1. Effects on Mass Balancing

To assess potential synergistic effects during co-pyrolysis, PO and SR were first subjected to individual pyrolysis experiments. Table 1 illustrates the relative product yields obtained from the separate pyrolysis of PO and SR at 600 °C. LDPE and PP generate high amounts of waxy products (≈90 wt%), small amounts of residue (<1 wt%), and moderate amounts of gaseous products (≈10 wt%). PS generated low quantities of gaseous products and residue, but in contrast, high amounts of liquid products (98.8 wt%). SR pyrolysis similarly yielded low amounts of gaseous products but produced substantial quantities of liquid/crystalline material (47.6 wt%) and solid residue (48.8 wt%). This behavior is attributed to the high filler content (30 wt% fumed silica) and the formation of silicone-based residues following side-chain elimination reactions. This observation is consistent with literature reports describing the formation of silica-rich residues during the pyrolysis of PDMS-based silicone rubbers [35,36]. For analytical consistency, the combined fraction of crystalline, liquid, and waxy products is classified as the liquid phase.

**Figure 1:**
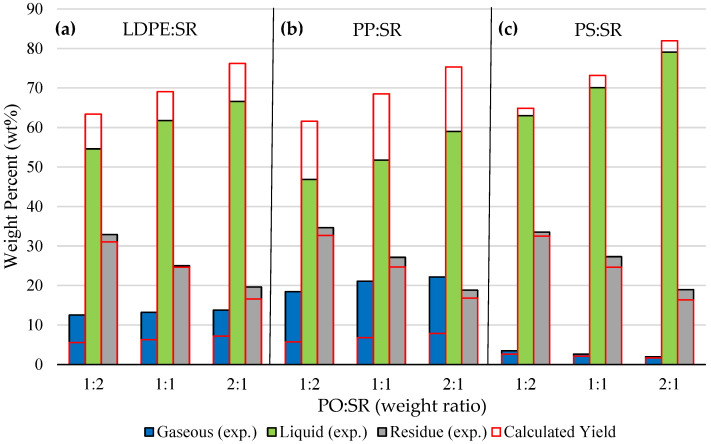
Experimental product distribution of LDPE:SR (**a**), PP:SR (**b**), and PS:SR (**c**) co-pyrolysis in different mass ratios. Calculated yields are based on pure polymer pyrolysis data.

Figure 1 illustrates the comparison between experimental and calculated product distributions derived from pure polymer pyrolysis data, obtained during the co-pyrolysis of PO and SR at varying mass ratios. In all cases, co-pyrolysis resulted in higher yields of gaseous products and solid residue than predicted, accompanied by a corresponding reduction in liquid yield. This effect becomes increasingly pronounced in mixtures containing a higher proportion of SR (66 wt%). This deviation could be attributed to intensified cracking reactions that favor the formation of lighter hydrocarbons under pyrolytic conditions. The phenomenon is likely driven by the distinct thermal behaviors and interactions of the polymer components. Polyolefins undergo melting during pyrolysis, whereas SR-based materials do not melt due to being chemically crosslinked [36], but instead become brittle and develop surface fissures, a phenomenon observed during pyrolysis experiments. The molten polymer may infiltrate these cracks, effectively increasing its residence time within the pyrolysis zone. This prolonged exposure promotes secondary thermal degradation, leading to the formation of lower molecular weight hydrocarbons. The effect is particularly pronounced in PP, whose pyrolysis products exhibit a high degree of substitution. These branched hydrocarbons are more volatile than their linear counterparts, resulting in a substantial increase in the gaseous fraction compared to LDPE. In contrast, PS primarily produces aromatic hydrocarbons that remain largely in the liquid phase. Although some cracking to smaller fragments occurs, this does not significantly enhance gaseous product formation. Prolonged exposure not only increases the gaseous fraction but also promotes solid residue formation, with all polymers exhibiting comparable behavior.

### 3.2. Analysis of Gaseous Products

**Figure 2:**
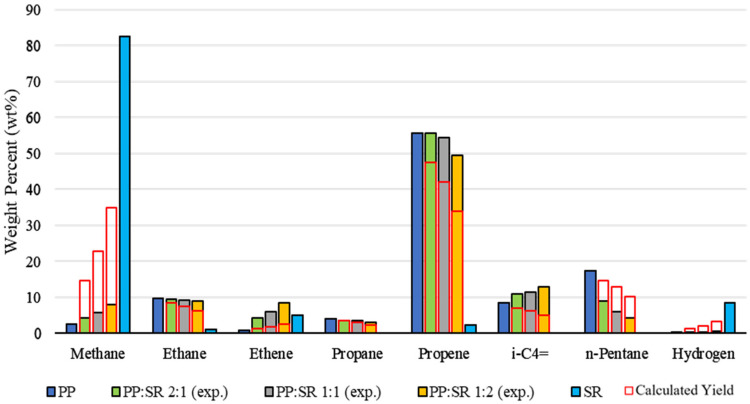
Comparison of experimental gaseous product composition during the pyrolysis of PP and SR. Calculated yields are based on pure polymer pyrolysis data.

The gas phase signatures of the individual polymers reveal very different degradation products. SR pyrolysis is characterized by an overwhelming formation of methane, accompanied by hydrogen and only minor amounts of short-chained olefins, reflecting the preferential cleavage of methyl substituents and the formation of silicon-rich residues. In contrast, polypropylene (PP) produces a gas mixture dominated by propene, consistent with β-scission of its tertiary carbon backbone, alongside notable amounts of C_5_ and smaller C_2_–C_3_ hydrocarbons. When PP is co-pyrolyzed with SR, the gas composition progressively shifts toward the profile typical of silicone degradation, but the shift is smaller than calculated. Methane and hydrogen increase only modestly, whereas certain hydrocarbons, particularly ethene and *iso*-butene, rise far more strongly than expected. The pronounced formation of branched olefins confirms the assumption that co-pyrolysis promotes secondary cracking reactions, leading to the formation of lighter hydrocarbons, evident by the rapid decrease in *n*-pentane. The gas composition and calculated gas composition of PP and SR mixtures are depicted in Figure 2.

**Figure 3:**
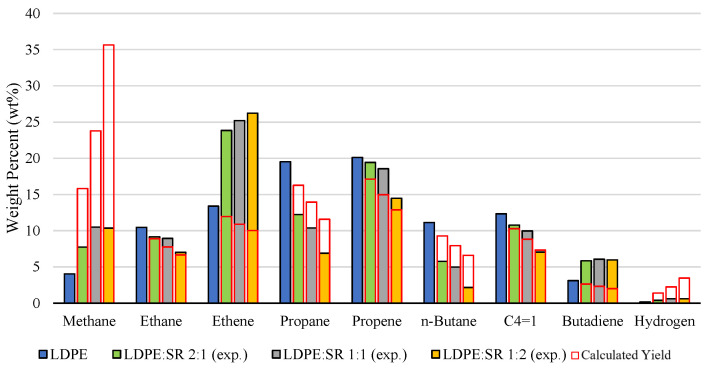
Comparison of experimental (exp.) gaseous product composition during the pyrolysis of LDPE and SR. Calculated yields are based on pure polymer pyrolysis data.

A similar pattern emerges for LDPE. While its pyrolysis typically yields a broad distribution of C_2_–C_4_ hydrocarbons, increasing SR content systematically enriches the gas phase in methane and, more strikingly, in ethene. The strong ethene enhancement, despite expectations of a decline, points to intensified chain scission and accelerated cracking toward the lighter olefins. Furthermore, the concurrent rise in 1,3-butadiene indicates that co-pyrolysis also reshapes the distribution of other light hydrocarbons, although the specific processes responsible for this increase remain uncertain. Other hydrocarbons generally follow expected dilution trends, though often at different rates, underscoring that the interaction between SR and polyolefins is subtle but sufficient to reshape the distribution of light gases. The main experimental and calculated gaseous products of LDPE and SR mixtures are depicted in Figure 3. Gaseous products only formed in minor amounts (<1 wt%), which follow the calculated trend, and are included in Figure S5. 

Overall, the deviations from calculated yields can be attributed to the addition of SR, which promotes the formation of lighter hydrocarbons and unsaturated species during co-pyrolysis and an overall increase in gaseous products. Consequently, the gaseous fraction becomes enriched in polyolefin-derived cracking products, while the dominant species still reflect the degradation pathways of the respective polyolefins, since no new compounds formed during co-pyrolysis could be detected. SR forms only minor amounts of gaseous products during pyrolysis; hence, the experimental yield differs greatly from the calculated yield for the main compounds. Overall, increased SR content produces significantly higher amounts of monomer than calculated, making it ideal for monomeric recovery. Due to the low amounts of gaseous products being formed during the pyrolysis of PS and PS:SR mixtures, no analysis of the gaseous products could be conducted.

### 3.3. Effect on Liquid Product Distribution

**Figure 4:**
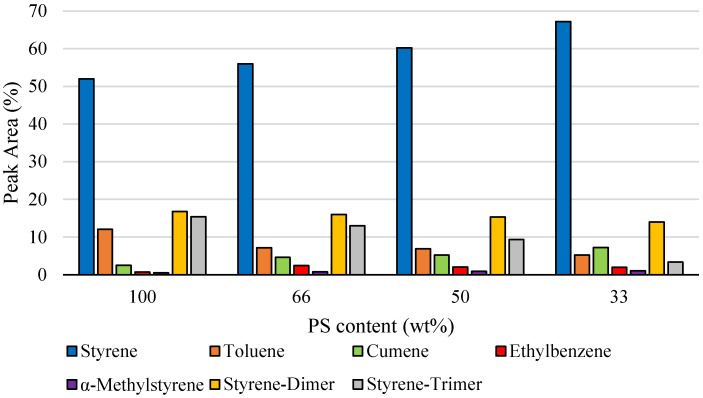
Quantitative analysis of main compounds generated during PS:SR co-pyrolysis (siloxanes excluded).

Co-pyrolysis of PS with increasing SR content markedly influences the distribution of liquid products, as demonstrated in Figure 4. The identified pyrolysis products are consistent with those reported in the literature; however, at comparable temperatures, a higher proportion of dimeric and trimeric species was observed in the present study. This effect is most likely attributable to the different operating conditions [37,38,39]. As the proportion of SR rises, the formation of low molecular weight compounds increases, while the yields of dimers and trimers decline. Styrene yield grows significantly from 52.0% at 100 wt% PS to 67.2% at 33 wt% PS, and α-methylstyrene shows a similar positive correlation, increasing from 2.5% to 7.2%. These trends indicate that SR promotes depolymerization reactions, favoring monomer and substituted monomer formation. Conversely, higher oligomers decrease substantially, with dimers falling from 16.8% to 14.0% and trimers from 15.4% to 3.4%. Minor aromatic species exhibit mixed behavior: toluene decreases from 12.0% to 5.2%, whereas ethylbenzene and cumene show slight increases. Overall, SR addition shifts PS pyrolysis toward lighter, monomeric products at the expense of oligomer formation, a feature that may benefit processes targeting monomer recovery.

**Figure 5:**
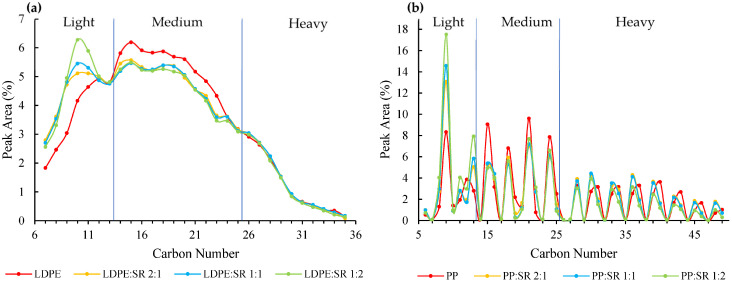
Product distribution of (**a**) LDPE:SR and (**b**) PP:SR mixtures. LDPE generates a broad spectrum of hydrocarbons spanning numerous chain lengths, whereas PP produces a narrow distribution dominated by C_3n_ species, accompanied by only small amounts of C_3n+1_ hydrocarbons.

The pyrolysis products of LDPE and PP were classified according to their carbon number, reflecting similarities in physicochemical properties, and they are summarized in Figure 5. Furthermore, the products were subdivided into three distinct fractions (light, medium, and heavy) based on their carbon number and their respective response to SR addition. Although PP produces significantly more highly substituted hydrocarbons than LDPE, the influence of SR addition on the fraction distribution remains similar for both polymers. The results are summarized in Table 2. The liquid and gaseous products obtained from the pyrolysis of the pure polymers are consistent with literature reports for LDPE [25,40,41] and PP [42,43,44] at comparable conditions. While the types of products identified and the predominant species are similar, differences in quantitative product yields are observed, most likely due to variations in operating conditions. Analysis of the liquid pyrolysis products of PP mixtures indicates that the addition of SR promotes the formation of light hydrocarbons (C_7_–C_13_), with their proportion increasing from 20.4% to 38.5%. The most abundant compound in this fraction, C_9_, rises markedly from 8.3% to 17.5%. In contrast, the yield of medium hydrocarbons (C_14_–C_25_) is higher for pure PP pyrolysis than for co-pyrolysis products, decreasing from 43.6% to 34.5% upon SR addition. The heavy fraction (>C_25_) shows no consistent trend, varying between 27.1% and 37.4%. Interestingly, in the heavy fraction of pure PP pyrolysis, C_3n+1_ compounds occur in greater abundance compared to C_3n_ species.

The co-pyrolysis of LDPE with SR exhibits a similar hydrocarbon profile to that of pure LDPE, with no new compounds detected. However, as observed for PP co-pyrolysis, SR addition shifts the product distribution toward shorter-chain hydrocarbons. The light fraction (C_7_–C_13_) increases from 25.9% to 32.8%, with the major component, C_10_, rising from 4.2% to 6.3%. The medium fraction (C_14_–C_25_) decreases from 58.8% to 52.3%, while the heavy fraction remains relatively unchanged, ranging from 14.9% to 15.6%. Overall, similar to PP co-pyrolysis, LDPE products shift toward lighter hydrocarbons, accompanied by a reduction in medium-chain compounds. In contrast, the pyrolysis of LDPE and PP does not exhibit substantial differences in the yield of higher hydrocarbons, whereas PS shows a pronounced decrease. It is important to note that siloxane-derived products were not included in this quantitative comparison, as the focus was placed on hydrocarbon-based species originating from the PO component.

### 3.4. Effect on Silicone Rubber Product Distribution

**Figure 6:**
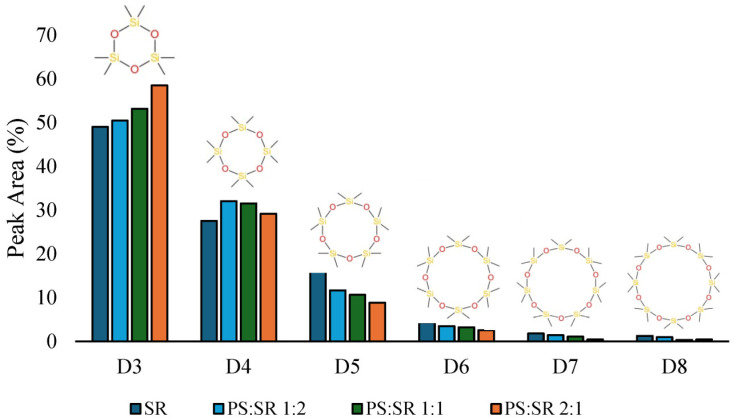
Yield of cyclic siloxanes in liquid pyrolysis products of PS:SR (hydrocarbons excluded).

During pyrolysis, SR predominantly decomposes into cyclic oligomers, with the major products being D_3_–D_5_, while larger cyclic siloxanes are formed only in minor quantities. The siloxane distribution observed during the pyrolysis of pure silicone rubber is consistent with literature data at comparable temperatures, with D_3_ identified as the dominant product and progressively lower yields observed for larger cyclic siloxanes [41,45]. During co-pyrolysis, no additional compounds are formed; however, the yield of siloxane compounds decreases relative to the calculated values, suggesting an increased formation of silicone-derived solid residue, as their contribution to the gaseous phase remains negligible. This effect is most pronounced during co-pyrolysis with LDPE, less evident with PP, and minimal in PS:SR mixtures. The distribution of individual cyclic siloxanes is also affected. The presence of polyolefins during pyrolysis enhances the formation of D_3_ while reducing the yield of higher cyclic siloxanes (D_5_–D_8_), whereas no clear trend is observed for D_4_. These observations indicate that, similar to hydrocarbon formation, co-pyrolysis favors the generation of smaller molecular species. This phenomenon is consistently observed across all investigated polyolefins (LDPE, PP, and PS). Enhanced D_3_ production under co-pyrolysis conditions streamlines separation steps, thereby increasing recovery efficiency and achievable yields. Figure 6 illustrates the siloxane yield for PS:SR mixtures, as LDPE and PP mixtures exhibit similar trends. 

Overall, the calculated siloxane yield in the liquid fraction aligns well with experimental measurements. As an example, for PS, this trend is evident across all PS:SR ratios. The experimental yields for siloxanes in the liquid product are 19.9% (1:2), 30.0% (1:1), and 48.5% (2:1), while the corresponding calculated yields are 19.2%, 32.6%, and 48.7%, respectively. The systematic decrease in the experimental values suggests that part of the SR does not convert into volatile siloxanes but instead contributes to the higher amount of solid residue. The GC-MS spectra and data of all liquid products are depicted in Figures S5–S18 and Tables S2–S4 of the Supplementary Material.

### 3.5. Mechanistic Interactions Between Polyolefins and Silicone Rubber

**Figure 7:**
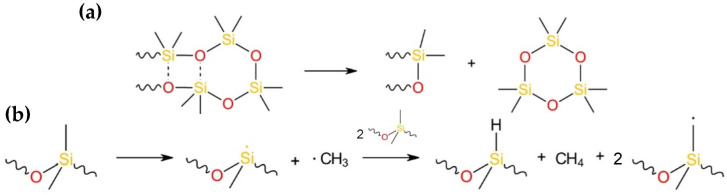
Formation of cyclic siloxanes through a cyclic transition state (**a**) and radical side chain elimination reaction (**b**) during PDMS degradation [17].

The thermal degradation pathways of polyolefins have been widely investigated and are predominantly governed by radical mechanisms. LDPE and PP degrade predominantly via free-radical chain reactions initiated by random C–C bond scission along the polymer backbone. The resulting macroradicals undergo β-scission, hydrogen abstraction, and radical recombination, producing mainly aliphatic hydrocarbons. While LDPE favors a broad distribution of linear and branched alkenes and alkanes due to secondary C–C scission, PP degrades more readily at tertiary carbon sites, often yielding higher fractions of low molecular weight olefins, such as propene [46,47]. In contrast, PS decomposes primarily via a radical depolymerization mechanism enabled by the stabilization of benzyl radicals by the aromatic ring. Secondary reactions produce alkyl-substituted aromatics, such as toluene and ethylbenzene. Hydrogen transfer reactions account for the production of dimer and trimer [48,49]. The principal degradation pathways of PDMS proceed either through a cyclic transition state or via radical chain scission accompanied by side-chain elimination (Figure 7) [17]. 

GC–MS and ^29^Si-NMR analyses show that co-pyrolysis of polyolefins with silicones does not generate additional liquid or gaseous products beyond those formed by the individual polymers, indicating that the dominant degradation pathways remain essentially unchanged. Silicone can generate reactive radicals at elevated temperatures through cleavage of the Si-C bond, leading to the formation of short-chained gaseous hydrocarbons [50]. The hydrocarbon radicals could take part in the degradation mechanism of polyolefins. However, radical formation is only minor, which is evident by the low amounts of gaseous products (3.6 wt%) during pure SR pyrolysis. An interaction of siloxane radicals with radicals generated from PO during degradation appears unlikely, as ^29^Si-NMR analysis revealed no additional signals across different mass ratios examined. This implies that strong mechanistic interactions between the polymers are most likely only minimal. The product distribution of the liquid fraction reveals that increasing SR content promotes the formation of lighter hydrocarbons and smaller siloxane rings. These significant shifts in the product distributions are, therefore, more plausibly attributed to differences in the thermal properties of SR and polyolefins, leading to an increased residence time rather than to significant mechanistic interactions during pyrolysis. NMR spectra of the liquid products are provided in Figures S19 and S20.

### 3.6. Recovery of Hexamethylcyclotrisiloxane (D_3_) from Liquid Pyrolysis Products

The isolation of D_3_ from mixtures containing various cyclic siloxanes together with hydrocarbons is challenging due to their chemical similarity. Pure siloxane mixtures are typically separated by distillation under ambient or atmospheric pressure; however, while several techniques aim to reduce siloxane content in complex matrices, such as pyrolysis oils [51,52,53], no established methods for selective isolation are reported in the literature.

D_3_ offers a unique opportunity for separation because of its relatively high melting point (64 °C) and low boiling point (134 °C). Hydrocarbons formed during polyolefin pyrolysis exhibit boiling points close to that of D_3_ but remain liquid at room temperature. Co-pyrolysis with polyolefins further enhances this potential, as the yield of D_3_ increases with higher polyolefin content. Nevertheless, the concurrent formation of lighter hydrocarbons complicates isolation. Co-pyrolysis products were initially fractionated by vacuum distillation. These fractions generally contain D_3_ along with hydrocarbons of similar boiling points, such as C_8_ and C_9_ aliphatic and olefinic species for LDPE, 2,4-dimethylheptadiene (137 °C), iso-C_9_ derivatives, and trimethylcyclohexane derivatives for PP and ethylbenzene (136 °C) and styrene (145 °C) for PS. Occasionally, larger cyclic siloxanes, such as D_4_ (174 °C), are also found in these fractions due to their high vapor pressure [54]. Recovery is more challenging for PS and PP systems because they produce larger quantities of hydrocarbons with boiling points close to D_3_ in comparison to LDPE, which is further enhanced during co-pyrolysis, although isolation remains feasible.

The solid part of D_3_-rich fractions (71.4% D_3_, 20.6% C_7–11_, 8.0% D_4_) obtained by vacuum distillation (200 mbar, 55 °C) of the liquid PP:SR co-pyrolysis products were separated from the remaining liquid phase by decantation, followed by purification using various solvents through washing, solvent-assisted filtration or recrystallization, and subsequent removal of the solvent under reduced pressure. All purification steps were performed at 4 °C to minimize the solubility of D_3_ in the hydrocarbon matrix and solvents, since purification at room temperature decreased the product yield significantly. For high recovery rates, D_3_, the used amount of solvent should be minimal; therefore, a solvent:distillation fraction weight ratio of 1:5 for washing and solvent-assisted filtration and a ratio of 1:4 for recrystallization for clear phase separation were chosen. Preliminary screening of solvent-to-distillate ratios outside the range of 1:5–1:4 with *n*-pentane showed either insufficient phase separation (1:6) or excessive D_3_ losses due to solubility at higher solvent volumes (1:3); therefore, only the optimized ratios were investigated systematically. As summarized in Table 3, solvent-assisted filtration consistently produced the highest purities and recovery rates, which can be attributed to the short solvent solid contact time that minimizes dissolution of D_3_ crystals. Ethanol and acetonitrile, in particular, afforded purities above 97% and high yields above 88%. Filtration without the addition of solvent yielded only moderate purities (79%), indicating the necessity of a solvent for the removal of liquid hydrocarbons. Washing resulted in moderate purities and yields, with more polar solvents such as ethanol, acetonitrile, and acetone performing best due to their limited ability to solubilize apolar D_3_, whereas DCM showed reduced purity and yield, likely because of good solubility properties towards D_3_. Recrystallization produced higher purities than washing but suffered from significantly reduced yields, as the larger solvent volumes increased D_3_ solubility and thus material loss. For highly polar solvents such as ethanol and acetonitrile, no separate liquid phase formed, preventing recrystallization entirely. Overall, the data reflects a clear polarity-dependent solubility trend in which polar solvents provide superior removal of hydrocarbon matrix and other siloxane compounds, while non-polar solvents such as *n*-pentane or diethyl ether dissolve D_3_ more readily and, therefore, reduce recovery. These results highlight solvent-assisted filtration as the most efficient and scalable purification strategy, combining high purity with high yield, while washing and recrystallization offer a simpler but less selective alternative.

Solvent-assisted filtration was also performed for a D_3_-rich distillation fraction containing high amounts of higher siloxane compounds derived from the LDPE:SR liquid co-pyrolysis product. The increase in the solvent:distillation fraction weight ratio to 1:4 led to higher purities (>99.5%) of the product, with losses of D_3_ yield. Furthermore, the drying conditions have been optimized, since longer drying times decrease D_3_ recovery due to its high vapor pressure. High concentrations of D_4_ in the distillation fraction could present a challenge for the isolation of D_3_. Due to its relatively high melting point (17 °C) [54], D_4_ remains solid at 4 °C and can only be separated in small quantities. Table 4 summarizes the yields and purities of solvent-assisted D_3_ fractions obtained after vacuum distillation.

Using vacuum distillation and solvent-assisted filtration with ethanol and optimized drying times on the pyrolysis product of LDPE:SR (1:1), 12.0 wt% D_3_ could be recovered (75.4% of theoretical yield determined by GC-MS) in high purity (>99.5%).

To evaluate D_3_ recovery with solvent-assisted filtration using ethanol (weight ratio of 1:4 solvent:distillation fraction), pure D_3_ was added in varying amounts to distilled pyrolysis products of LDPE, PP, and PS. Recovery was successful up to a D_3_ content of ≥40 wt% within a hydrocarbon matrix of ≤60 wt%. At higher hydrocarbon contents, crystallization at 4 °C did not occur, rendering purification via prior crystallization impossible. Although recovery remains feasible at D_3_ concentrations as low as 40 wt%, the yield decreases sharply when the hydrocarbon fraction exceeds 40 wt% due to increased solubility of D_3_ in the hydrocarbon matrix, which limits its separation. The recovery yields of D_3_ mixed with a distilled LDPE fraction under these conditions are summarized in Table 5. The mixture with distilled fractions of PP and PS indicated only negligible differences in D_3_ recovery yield or crystallization.

The recovery of D_3_ with the methods discussed is only possible due to its relatively high melting point. In contrast, D_4_ (m.p. 17 °C, b.p. 174 °C) could not be separated using the same technique, as no crystallization was observed at 4 °C, even in distillation fractions containing approximately 90% D_4_. Nevertheless, separation of D_4_ may be technically feasible at lower temperatures, where crystallization could occur, given that hydrocarbons with similar boiling points exhibit significantly lower melting points (e.g., C_10_H_32_: m.p. −30 °C, b.p. 174 °C; α-methylstyrene: m.p. −23 °C, b.p. 166 °C). For cyclic siloxanes larger than D_4_, such as D_5_ (m.p. −38 °C, b.p. 210 °C), isolation by crystallization alone appears highly unlikely due to the substantial gap between boiling and melting points, combined with the relatively higher melting points of hydrocarbons in the same boiling range (e.g., C_12_H_26_: m.p. −10 °C, b.p. 216 °C) [54].

## 4. Conclusions

Pyrolysis of polyolefins (LDPE, PP, PS) and silicone rubber mixtures was conducted in a laboratory-scale apparatus to evaluate product composition, synergistic interactions, and the potential for siloxane recovery. Co-pyrolysis of polyolefins with silicone rubber exhibited clear synergistic effects, characterized by a reduction in liquid product yield and an increase in gaseous products and solid residue with rising silicone rubber content. This effect was most pronounced for PP and marginal for PS. The gaseous fraction during the co-pyrolysis of silicone with LDPE and PP deviated from predictions, calculated from the pure polymer pyrolysis data, showing a higher proportion of lighter hydrocarbons. Chromatographic analysis of liquid products revealed no formation of new compounds; however, an increased presence of lighter hydrocarbons and smaller cyclic siloxanes was observed, particularly at higher silicone concentrations. These trends are attributed to the distinct thermal properties of the polymers, while molecular-level interactions during degradation appear to be only minimal. Silicone rubbers do not melt but become brittle, allowing molten polyolefins to penetrate surface cracks, thereby prolonging the residence time and promoting secondary cracking.

Recovery of the main product of silicone pyrolysis, hexamethylcyclotrisiloxane (D_3_), was demonstrated to be feasible during the co-pyrolysis of polyolefins and silicone rubber. Solvent-assisted filtration at 4 °C for the distillation fraction containing at least 40 wt% D_3_ was the most promising method, with polar solvents, like ethanol or acetonitrile, yielding high purity and significant quantities. Other methods, like washing or recrystallization, exhibit a significant decrease in purity and yield. While recovery of D_4_ may be possible at lower temperatures, isolation of larger cyclic siloxanes is unlikely with the methods discussed due to their low melting points relative to hydrocarbons in the same boiling range.

## Figures and Tables

**Table 1:** Mass balancing of pure polymer pyrolysis for LDPE, PP, PS, and SR at 600 °C.

	Gaseous	Liquid	Residue
	wt%	wt%	wt%
LDPE	9.02 ± 0.21	90.59 ± 0.18	0.39 ± 0.05
PP	10.35 ± 0.26	88.93 ± 0.28	0.72 ± 0.03
PS	0.71 ± 0.04	98.83 ± 0.06	0.46 ± 0.04
SR	3.59 ± 0.09	47.64 ± 0.21	48.76 ± 0.28

**Table 2:** Product distribution of LDPE/PP:SR liquid co-pyrolysis products classified into light, medium, and heavy fractions according to hydrocarbon chain length (siloxanes excluded).

Polymer Ratio (wt%)		Peak Area (%)		
LDPE	SR	Light (C_7_–C_13_)	Medium (C_14_–C_25_)	Heavy (>C_25_)
100	0	25.9	62.0	12.1
66	34	31.1	56.6	12.3
50	50	31.4	56.1	12.5
34	66	32.8	55.4	11.8
**PP**	**SR**	**Light (C_7_–C_13_)**	**Medium (C_14_–C_25_)**	**Heavy (>C_25_)**
100	0	20.4	43.5	36.1
66	34	27.6	34.9	37.5
50	50	30.0	34.0	36.0
34	66	38.5	33.8	27.7

**Table 3:** Yield and purity of recovered D_3_ from a hydrocarbon-rich D_3_ distillation fraction obtained from the liquid product of PP:SR (1:1) at 200 mbar and 55 °C by washing, recrystallization, and solvent-assisted filtration.

	Washing		Solvent-Assisted Filtration		Recrystallization	
	Purity	Yield (Based on D_3_)	Purity	Yield (Based on D_3_)	Purity	Yield (Based on D_3_)
	(%)	(%)	(%)	(%)	(%)	(%)
*n*-Pentane	89.7	79.5	88.8	82.8	90.3	43.5
Dichloromethane	62.8	54.2	91.9	68.0	77.2	37.6
Diethyl ether	77.7	73.3	93.5	81.4	85.0	42.6
Acetone	79.6	84.2	94.3	86.6	86.0	46.3
Acetonitrile	70.4	83.5	97.1	88.8	no phase separation	
Ethanol	84.8	81.1	98.6	96.5	no phase separation	
No solvent	-	-	78.8	-	-	-

**Table 4:** Yields and composition of solvent-assisted filtration of siloxane-rich D_3_ distillation fractions obtained from the liquid product of LDPE:SR (1:1) at 200 mbar and 55 °C.

	Drying	Composition	Yield (Based on D_3_) (wt%)
Distillation fraction	-	63.8% D_3_, 14.7% C_6_–C_12_, 21.2% D_4_, 0.26% D_5_, 0.1% D_6_	-
*n*-Pentane	4 mbar, 5 min	99.9% D_3_, 0.1% D_4_	52.0
Dichloromethane	4 mbar, 5 min	99.9% D_3_, 0.1% D_4_	42.0
Diethyl ether	4 mbar, 5 min	99.9% D_3_, 0.1% D_4_, 0.1% C_8_	50.4
Acetone	4 mbar, 5 min	99.8% D_3_, 0.2% D_4_	59.9
Acetonitrile	4 mbar, 10 min	99.8 D_3_, 0.1% C_6_–C_12_, 0.1% D_4_	77.4
Ethanol	4 mbar, 10 min	99.9% D_3_, 0.1% D_4_ and D_5_	87.1
Only filtration	4 mbar, 10 min	86.0% D_3_, 10.0% D_4_, 3.7% C_6_–C_12_, 0.3% D_5_	-

**Table 5:** Recovery yield of D_3_ from mixtures of D_3_ and distilled LDPE pyrolysis fractions (200 mbar, 55 °C).

D_3_	LDPE Distilled Fraction	Yield (Based on D_3_)
(wt%)	(wt%)	(wt%)
89.8	10.2	97.4
79.6	20.4	91.0
69.0	31.0	85.9
60.8	39.2	83.2
50.1	49.9	52.5
40.5	59.5	26.6
<40.5	>59.5	no recovery possible

## Data Availability

The original contributions presented in this study are included in the article/Supplementary Materials. Further inquiries can be directed to the corresponding author.

## Supplementary Materials

The following supporting information can be downloaded at: https://www.mdpi.com/article/10.3390/polym18080989/s1, Figure S1: TGA of LDPE, Figure S2: TGA of PP, Figure S3: TGA of PS, Figure S4: TGA of silicone rubber, Figure S5: Comparison of experimental (exp.) and calculated (calc.) gaseous product composition during the pyrolysis of LDPE and silicone rubber, Figure S6: LDPE:silicone rubber 1:2 liquid pyrolysis product GC-MS data, Figure S7: LDPE:silicone rubber 1:1 liquid pyrolysis product GC-MS data, Figure S8: LDPE:silicone rubber 2:1 liquid pyrolysis product GC-MS data, Figure S9: LDPE liquid pyrolysis product GC-MS data, Figure S10: PP:silicone rubber 1:2 liquid pyrolysis product GC-MS data, Figure S11: PP:silicone rubber 1:1 liquid pyrolysis product GC-MS data, Figure S12: PP:silicone rubber 2:1 liquid pyrolysis product GC-MS data, Figure S13: PP liquid pyrolysis product GC-MS data, Figure S14: PS:silicone rubber 1:2 liquid pyrolysis product GC-MS data, Figure S15: PS:silicone rubber 1:1 liquid pyrolysis product GC-MS data, Figure S16: PS:silicone rubber 2:1 liquid pyrolysis product GC-MS data, Figure S17: PS liquid pyrolysis product GC-MS data, Figure S18: Silicone rubber liquid pyrolysis product GC-MS data, Figure S19: 29Si-NMR spectra of liquid LDPE:silicone rubber co-pyrolysis product, Figure S20: 29Si-NMR spectra of liquid silicone rubber co-pyrolysis product, Table S1: Peak degradation temperature (TP) of PO and SR, Table S2: Hydrocarbon composition of liquid LDPE:silicone rubber co-pyrolysis products, Table S3: Hydrocarbon distribution of liquid PP:silicone rubber co-pyrolysis products, Table S4: Siloxane distribution of liquid polyolefin:silicone rubber co-pyrolysis products.

## Abbreviations

The following abbreviations are used in this manuscript:

HDPE	High-density polyethylene
LDPE	Low-density polyethylene
LLDPE	Linear low-density polyethylene
PDMS	Polydimethylsiloxane
PO	Polyolefin
PP	Polypropylene
PS	Polystyrene
SR	Silicone rubber
UHMW	Ultra-high molecular weight
D_x_	Cyclic siloxanes, whereas x is the number of repeating -Si(CH_3_)_2_O- units
C_y_	General formula for hydrocarbons; here: linear hydrocarbons if not stated otherwise
DCM	Dichloromethane
b.p.	Boiling point
m.p.	Melting point
FID	Flame ionization detector
FTIR	Fourier transform infrared spectroscopy
GC	Gas chromatography
MS	Mass spectrometry
NMR	Nuclear magnetic resonance
TCD	Thermal conductivity detector
