# Supplementary material for: Co-Pyrolysis of Polyolefins and Silicone Rubber: Effects on Mass Balancing, Product Distribution, and Potential Siloxane Recovery
Source: Polymers (Basel). 2026 Apr 18;18(8):989. doi: 10.3390/polym18080989 (PMC13120016; doi:10.3390/polym18080989)
Supplement: Supplementary file 1 [file polymers-18-00989-s001.zip › polymers-4251983-supplementary.pdf]

Supplementary Material

for

Co-Pyrolysis of Polyolefins and Silicone Rubber: Effects on Mass Balancing, Product Distribution and potential Siloxane Recovery

Lukas Eigenschink[1,2*], Wolfgang Eder[3], Matthias Mastalir[3], Michael Harasek[2], Christian Paulik[1]

[a]*Johannes Kepler University Linz, Institute for Chemical Technology of Organic Materials, Altenberger Straße 69, 4040 Linz, Austria*
[b] *TU Wien, Institute of Chemical, Environmental and Bioscience Engineering, Getreidemarkt 9/166, A-1060, Vienna, Austria*
[c]*OMV Downstream GmbH, Mannswörther Straße 28, 2320 Schwechat, Austria*

Figure S1: TGA of LDPE 2
Figure S2: TGA of PP 3
Figure S3: TGA of PS 3
Figure S4: TGA of Silicone Rubber 4
Figure S5: Comparison of experimental (exp.) and calculated (calc.) gaseous product composition during the pyrolysis of LDPE and silicone rubber 5
Figure S6: LDPE:Silicone rubber 1:2 liquid pyrolysis product GC-MS data 8
Figure S7: LDPE:Silicone rubber 1:1 liquid pyrolysis product GC-MS data 9
Figure S8: LDPE:Silicone rubber 2:1 liquid pyrolysis product GC-MS data 9
Figure S9: LDPE liquid pyrolysis product GC-MS data 10
Figure S10: PP:Silicone rubber 1:2 liquid pyrolysis product GC-MS data 11
Figure S11: PP:Silicone rubber 1:1 liquid pyrolysis product GC-MS data 11
Figure S12: PP:Silicone rubber 2:1 liquid pyrolysis product GC-MS data 12
Figure S13: PP liquid pyrolysis product GC-MS data 12
Figure S14: PS:Silicone rubber 1:2 liquid pyrolysis product GC-MS data 13
Figure S15: PS:Silicone rubber 1:1 liquid pyrolysis product GC-MS data 13
Figure S16: PS:Silicone rubber 2:1 liquid pyrolysis product GC-MS data 14
Figure S17: PS liquid pyrolysis product GC-MS data 14
Figure S18: Silicone rubber liquid pyrolysis product GC-MS data 15
Figure S19: ^{29}Si-NMR spectra of liquid LDPE:Silicone rubber co-pyrolysis product 15
Figure S20: ^{29}Si-NMR spectra of liquid silicone rubber co-pyrolysis product 16

Table S1: Peak degradation temperature (T_P) of PO and SR 4
Table S2: Hydrocarbon composition of liquid LDPE:Silicone rubber co-pyrolysis products 6
Table S3: Hydrocarbon distribution of liquid PP:Silicone rubber co-pyrolysis products 6

* Corresponding author
E-Mail address: lukas.eigenschink@jku.at

Table S4: Siloxane distribution of liquid polyolefin:silicone rubber co-pyrolysis products 7

Thermogravimetric Analysis (TGA)

Thermogravimetric analysis (TGA) of the polymers were recorded using a Perkin Elmer – TGA 8000 at a temperature range from 25 – 900 °C (heating rate of 15 °C min^{-1}) in N_2-atmosphere. An important parameter for TGA analysis is the maximum degradation temperature (T_P), which is defined as the temperature at which the maximum rate of mass loss occurs.

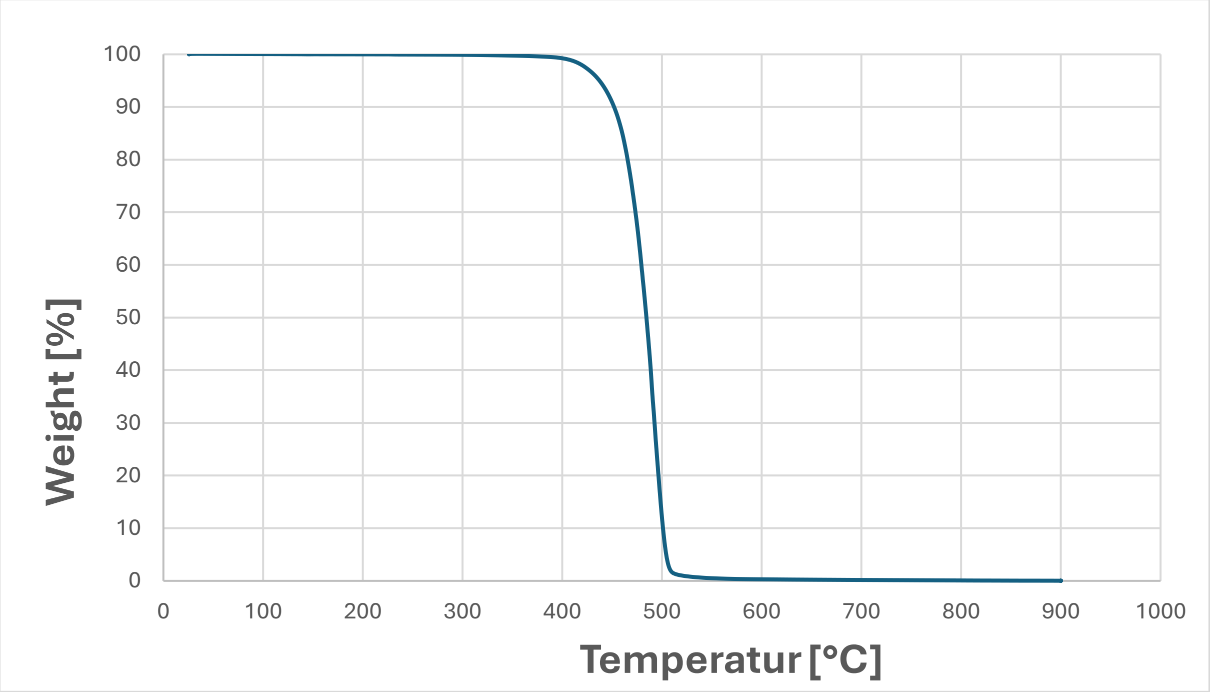

Figure S1: TGA of LDPE

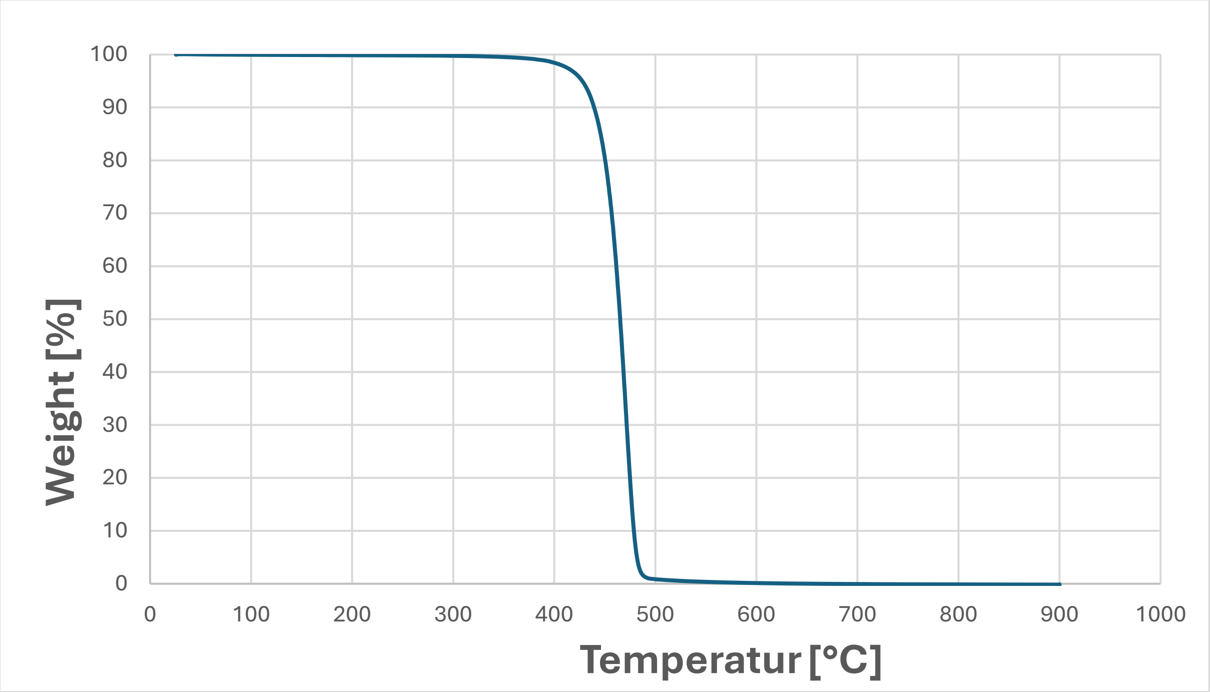

Figure S2: TGA of PP

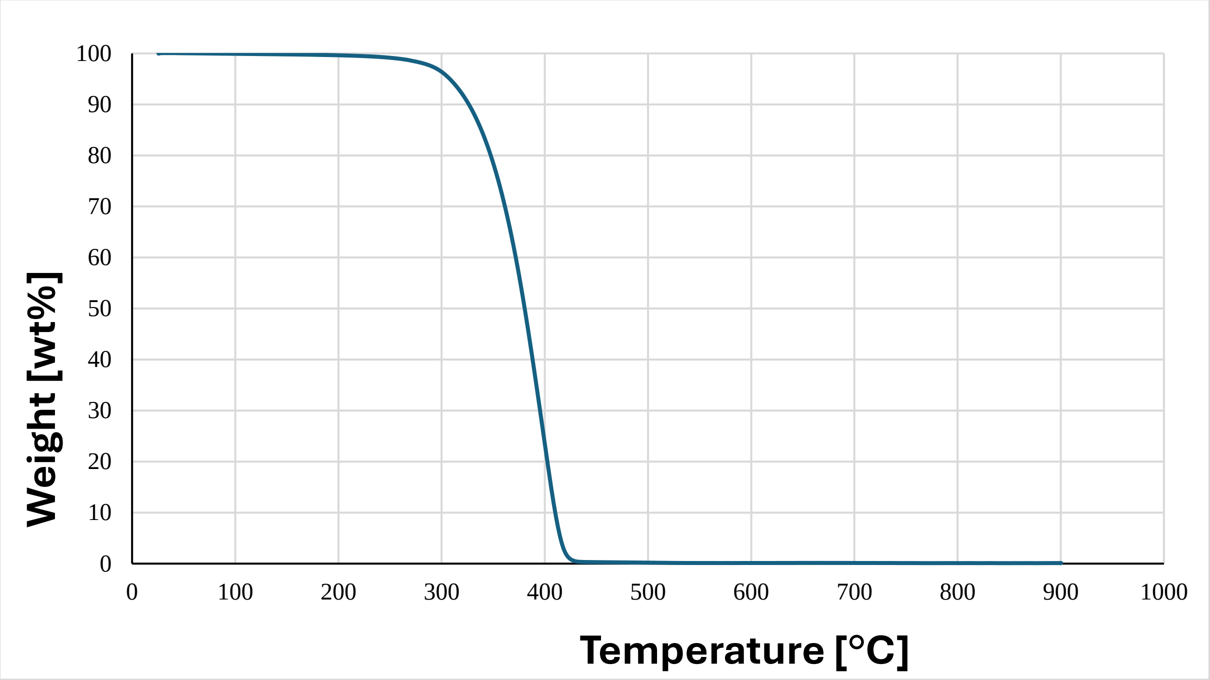

Figure S3: TGA of PS

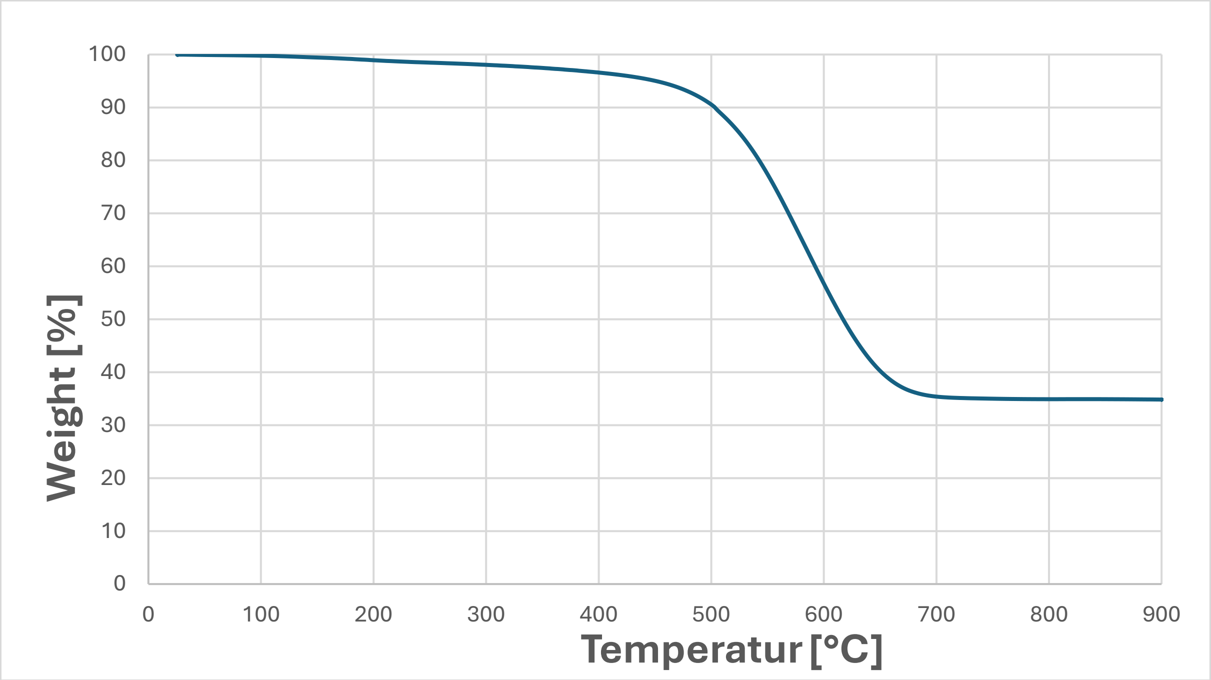

Figure S4: TGA of silicone rubber

Table S1: Peak degradation temperature (T_P) of PO and SR

		LDPE	PP	PS	SR
T_P	°C	482	470	423	563

a

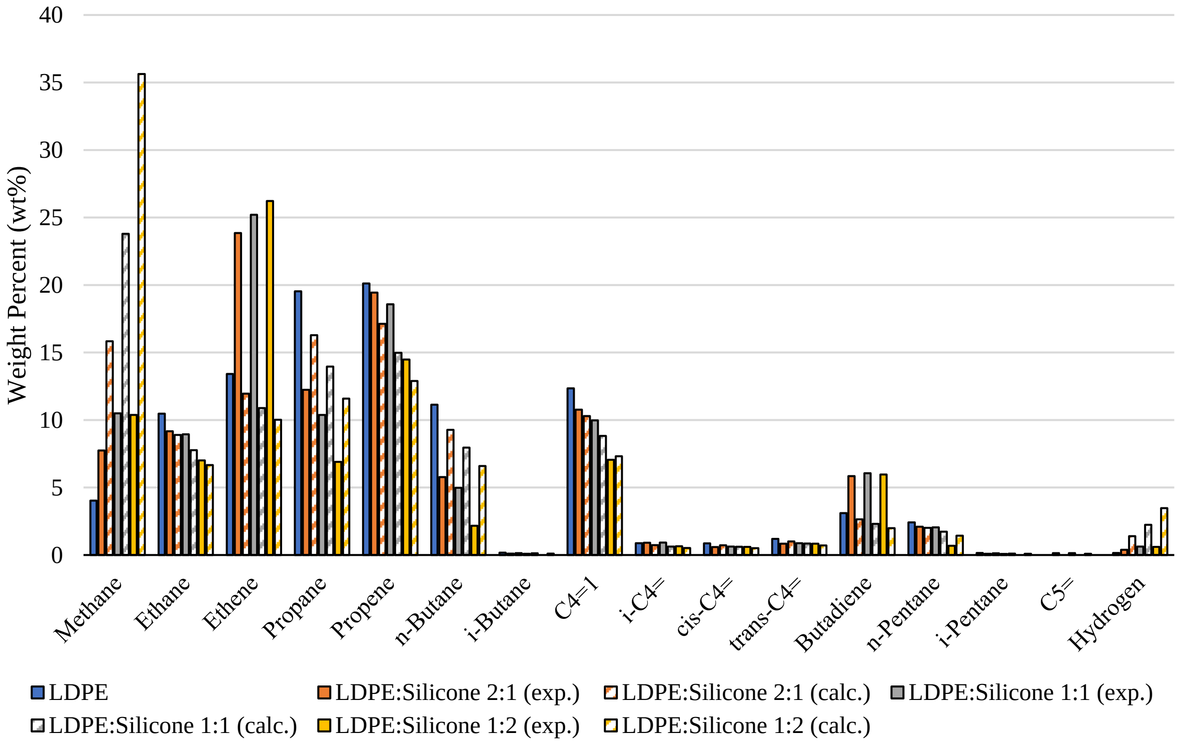

Figure S5: Comparison of experimental (exp.) and calculated (calc.) gaseous product composition during the pyrolysis of LDPE and silicone rubber

Table S2: Hydrocarbon composition of liquid LDPE:silicone rubber co-pyrolysis products

Carbon Number	Retention Time (min)	Peak Area (%)			
		LDPE	LDPE:Silicone rubber 2:1	LDPE:Silicone rubber 1:1	LDPE:Silicone rubber 1:2
7	2.246	1.83	2.79	2.70	2.56
8	3.215	2.46	3.61	3.54	3.31
9	4.358	3.04	4.72	4.81	4.95
10	5.514	4.16	5.11	5.45	6.28
11	6.623	4.64	5.11	5.31	5.89
12	7.664	4.92	5.00	4.87	5.01
13	8.643	4.81	4.79	4.75	4.81
14	9.562	5.81	5.45	5.19	5.25
15	10.429	6.19	5.57	5.46	5.49
16	11.252	5.91	5.33	5.27	5.23
17	12.031	5.83	5.22	5.25	5.20
18	12.769	5.87	5.38	5.39	5.26
19	13.473	5.69	5.37	5.35	5.17
20	14.146	5.60	4.96	5.06	5.03
21	14.789	5.17	4.54	4.57	4.54
22	15.405	4.84	4.33	4.23	4.16
23	15.997	4.33	3.65	3.59	3.47
24	16.592	3.60	3.61	3.60	3.46
25	17.133	3.18	3.17	3.12	3.09
26	17.653	2.91	3.03	3.04	2.98
27	18.153	2.63	2.71	2.70	2.68
28	18.649	2.13	2.23	2.24	2.08
29	19.187	1.52	1.54	1.53	1.49
30	19.796	0.87	0.87	0.93	0.84
31	20.500	0.66	0.65	0.64	0.61
32	21.326	0.55	0.53	0.55	0.47
33	22.316	0.35	0.37	0.41	0.35
34	23.513	0.35	0.28	0.29	0.21
35	24.993	0.15	0.08	0.17	0.13

Table S3: Hydrocarbon distribution of liquid PP:silicone rubber co-pyrolysis products

Carbon Number	Peak Area (%)			
	PP	PP:Silicone rubber 2:1	PP:Silicone rubber 1:1	PP:Silicone rubber 1:2
6	0.54	1.00	0.98	0.72
7	0.10	0.10	0.10	0.10
8	1.30	2.73	2.98	4.04
9	8.32	13.06	14.56	17.53
10	1.41	0.89	0.91	0.98
11	1.93	2.68	2.81	4.05
12	3.86	1.96	1.72	2.98
13	2.79	5.04	5.83	7.92

14	0.11	0.12	0.12	0.13
15	9.05	5.25	5.37	4.94
16	3.15	3.90	4.39	4.04
17	0.13	0.13	0.14	0.14
18	6.80	5.92	5.30	5.61
19	2.18	0.68	0.30	0.24
20	1.38	1.66	1.19	1.05
21	9.60	7.01	7.19	7.67
22	0.76	2.77	2.69	3.12
23	0.09	0.11	0.10	0.11
24	7.85	5.95	6.18	6.60
25	2.51	1.53	1.07	0.89
26	0.06	0.07	0.07	0.08
27	0.10	0.11	0.10	0.12
28	3.27	3.91	3.71	3.05
29	0.09	0.08	0.09	0.07
30	2.72	4.27	4.43	3.92
31	3.15	2.00	1.80	1.52
32	0.08	0.06	0.05	0.07
33	2.50	3.27	3.51	2.92
34	3.15	2.92	2.53	1.75
35	0.11	0.11	0.11	0.11
36	2.53	4.29	4.11	3.15
37	3.29	2.01	1.90	1.39
38	0.08	0.08	0.08	0.08
39	2.47	3.66	3.53	2.44
40	3.63	1.67	1.60	1.18
41	0.07	0.07	0.07	0.07
42	1.66	2.27	2.17	1.44
43	2.68	1.44	1.36	1.06
44	0.05	0.05	0.05	0.05
45	0.94	1.84	1.67	0.94
46	1.65	0.76	0.68	0.38
47	0.02	0.02	0.02	0.02
48	0.70	1.78	1.63	1.00
49	1.02	0.70	0.70	0.30

Table S4: Siloxane distribution of liquid polyolefin:silicone rubber co-pyrolysis products

	Peak Area (%)								
	**PS:Silicone 1:2**	**PS:Silicone 1:1**	**PS:Silicone 2:1**	**PP:Silicone 1:2**	**PP:Silicone 1:1**	**PP:Silicone 2:1**	**LDPE:Silicone 1:2**	**LDPE:Silicone 1:1**	**LDPE:Silico 2:1**
**D_3**	50.44	53.18	58.50	50.03	53.33	58.18	49.99	52.89	57.99
**D_4**	32.04	31.52	29.18	31.53	31.39	28.72	32.04	31.52	29.18
**D_5**	11.66	10.64	8.85	11.42	10.84	9.01	11.33	10.74	8.78
**D_6**	3.48	3.22	2.57	3.44	3.33	2.93	3.22	3.02	2.09
**D_7**	1.42	1.10	0.45	1.36	1.01	0.37	1.33	1.01	0.40
**D_8**	0.95	0.35	0.45	0.95	0.44	0.39	0.87	0.30	0.29

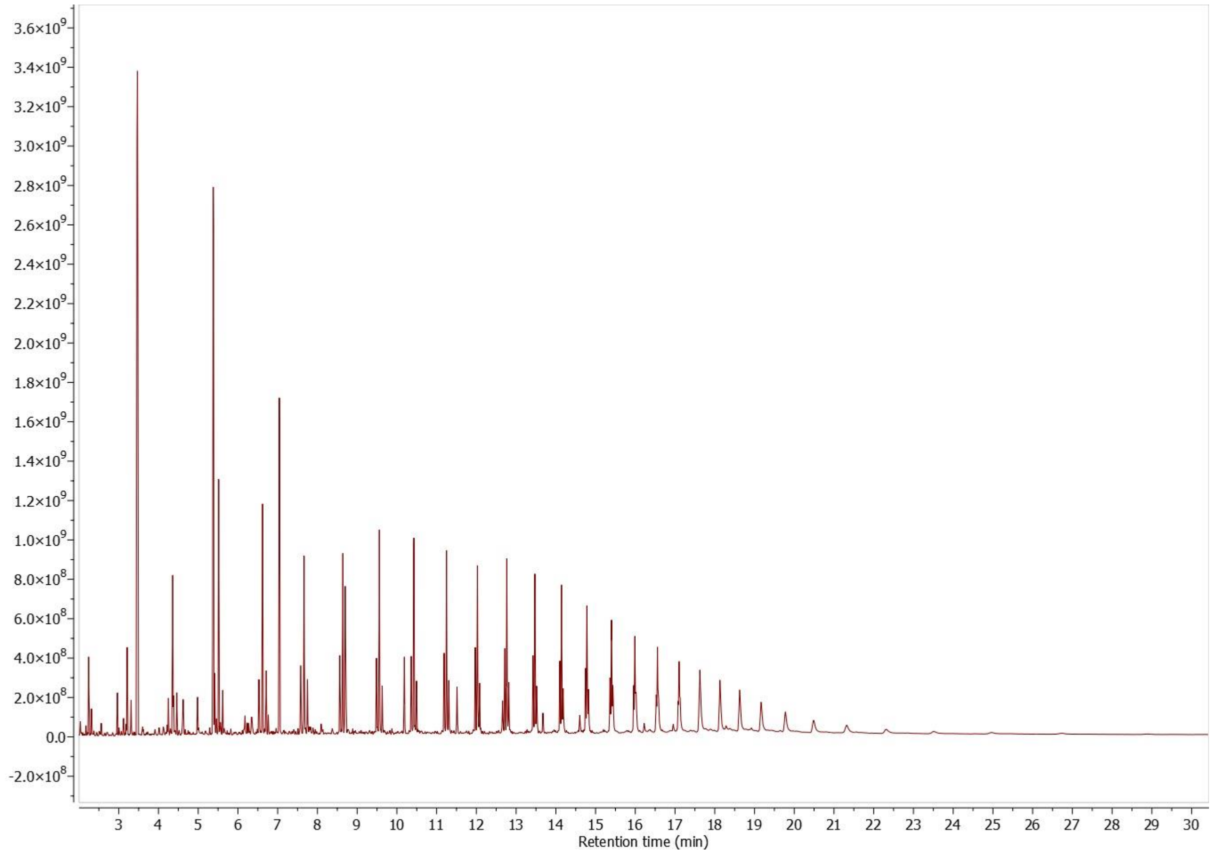

Figure S6: LDPE:silicone rubber 1:2 liquid pyrolysis product GC-MS data

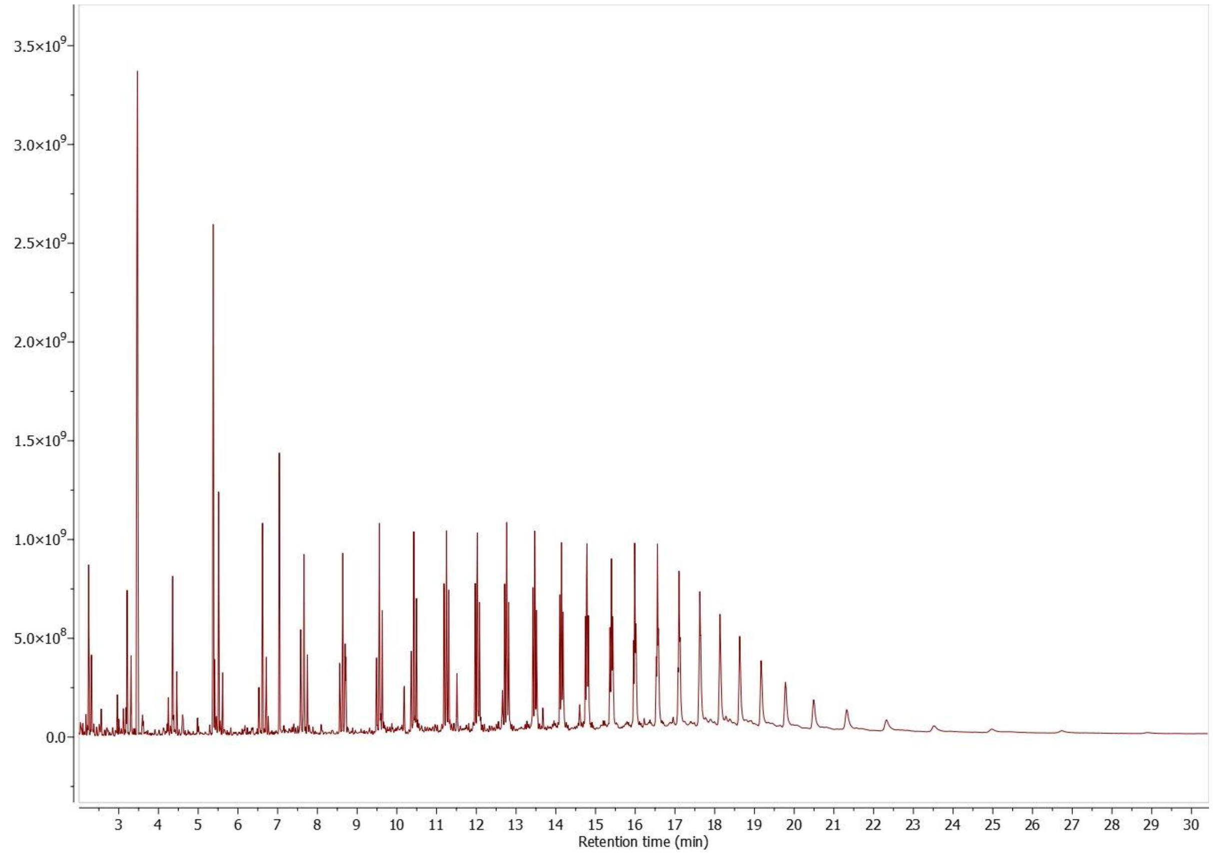

Figure S7: LDPE:silicone rubber 1:1 liquid pyrolysis product GC-MS data

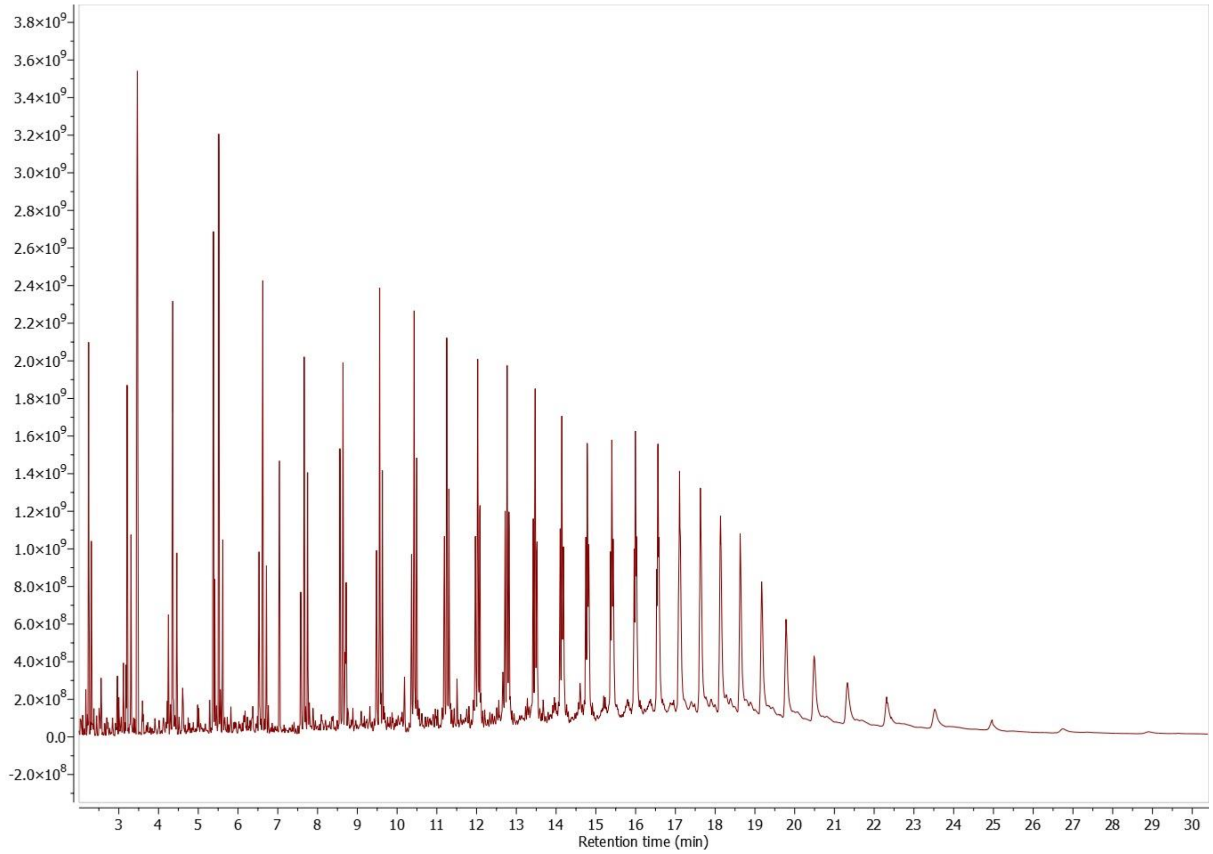

Figure S8: LDPE:silicone rubber 2:1 liquid pyrolysis product GC-MS data

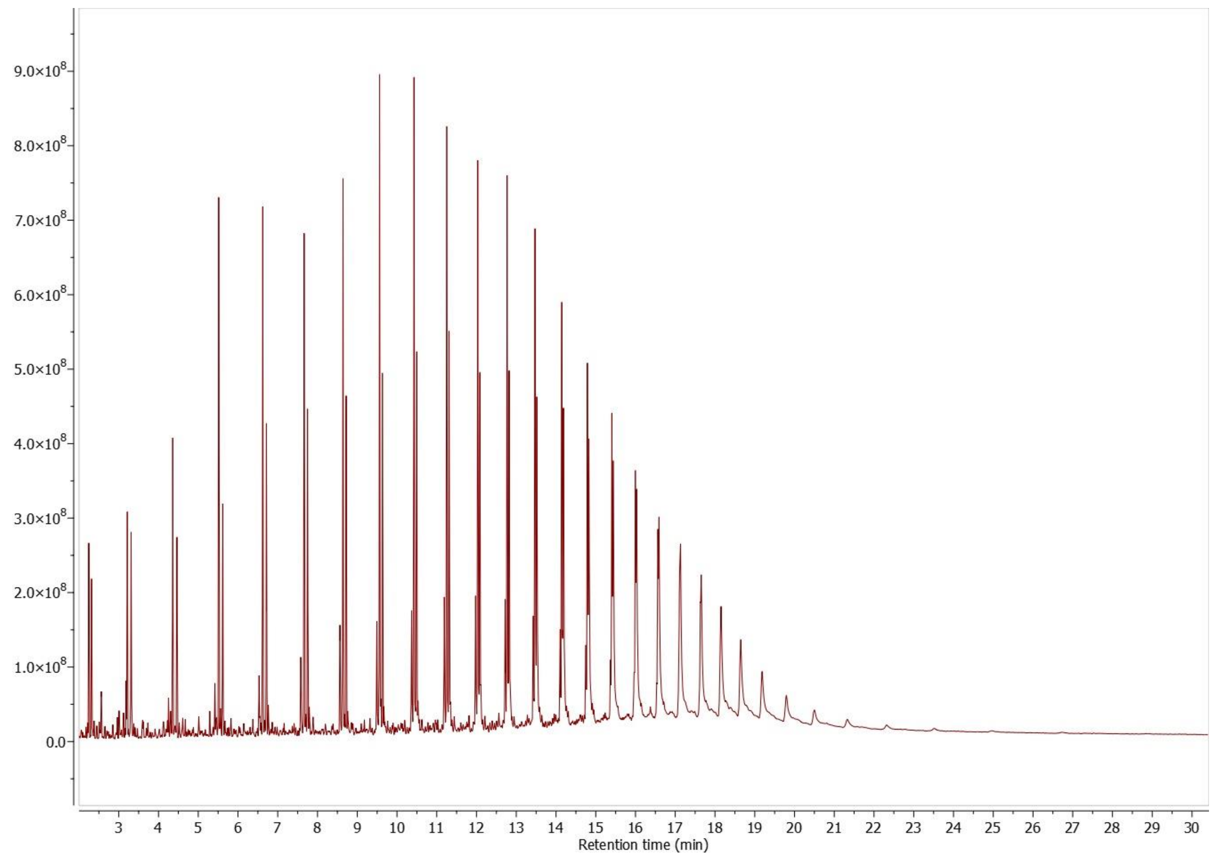

Figure S9: LDPE liquid pyrolysis product GC-MS data

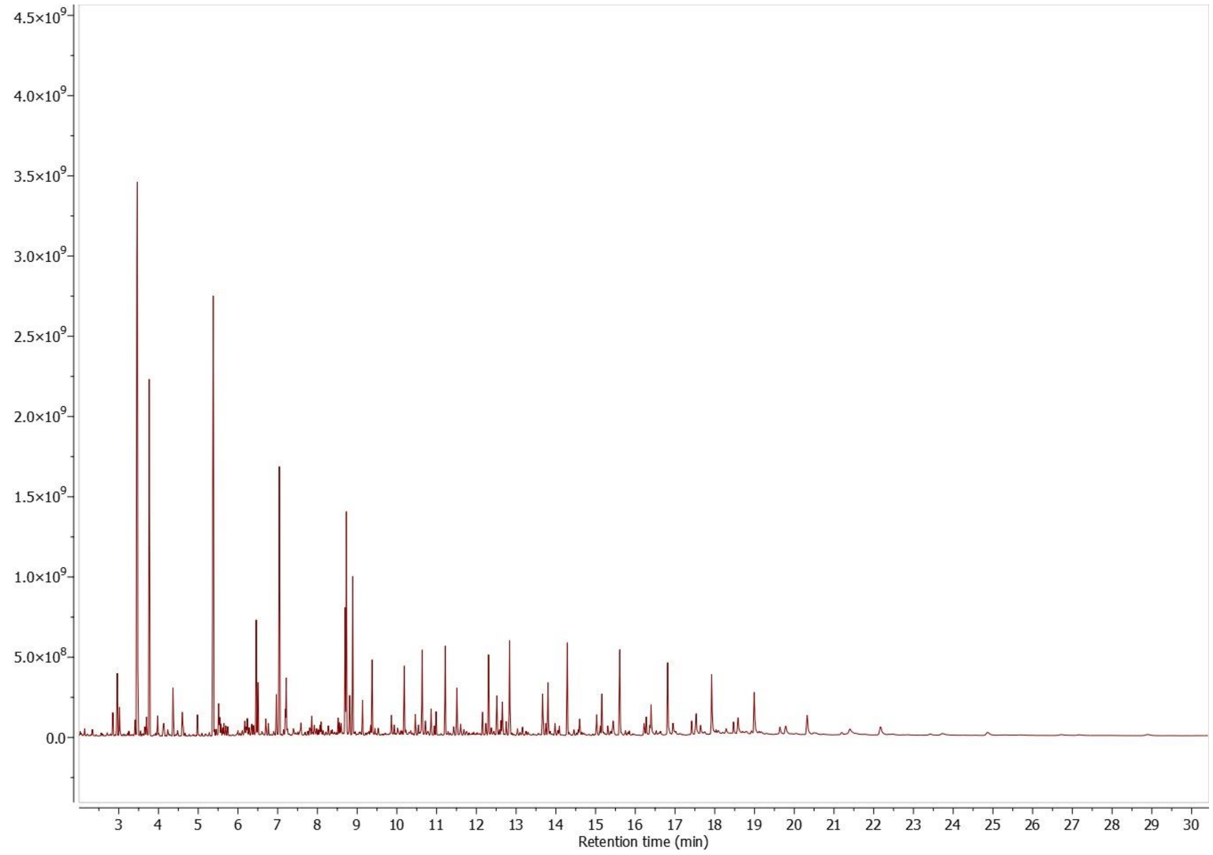

Figure S10: PP:silicone rubber 1:2 liquid pyrolysis product GC-MS data

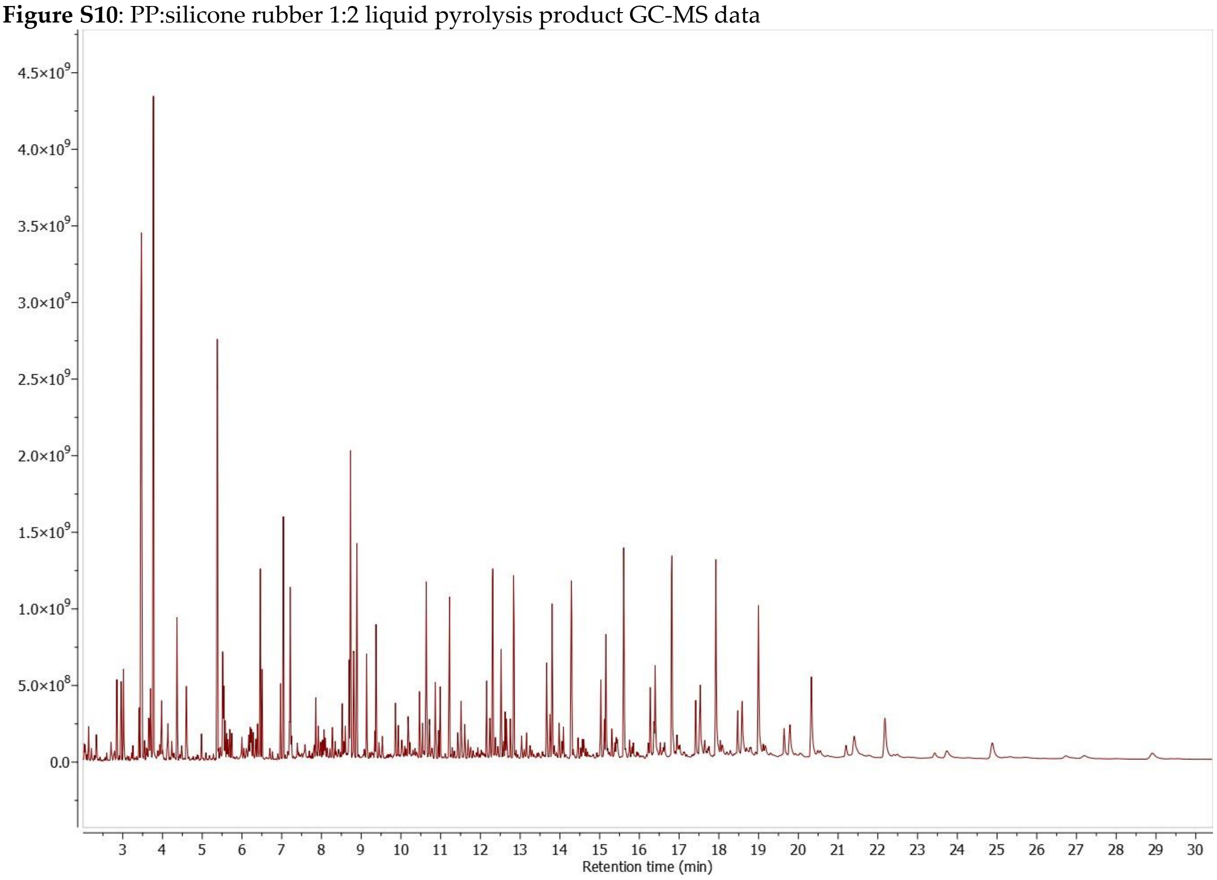

Figure S11: PP:silicone rubber 1:1 liquid pyrolysis product GC-MS data

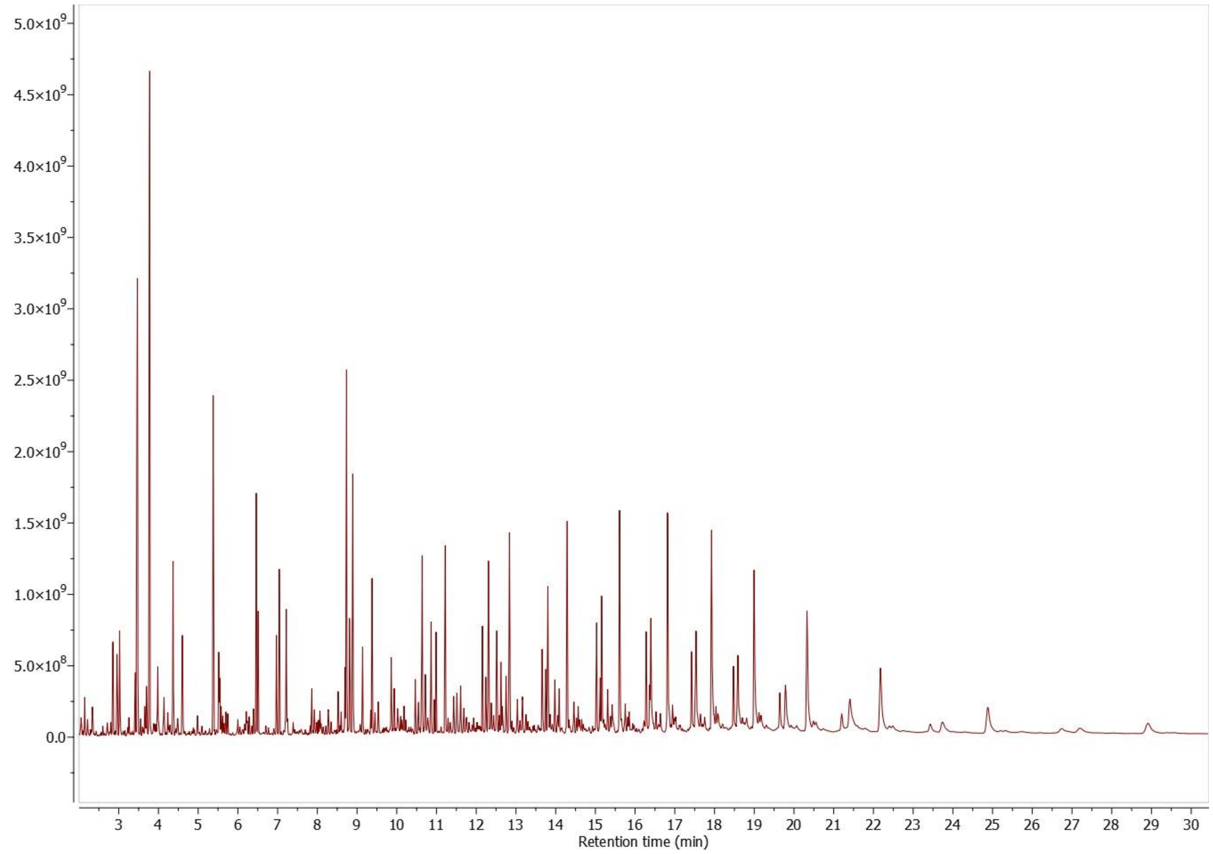

Figure S12: PP:silicone rubber 2:1 liquid pyrolysis product GC-MS data

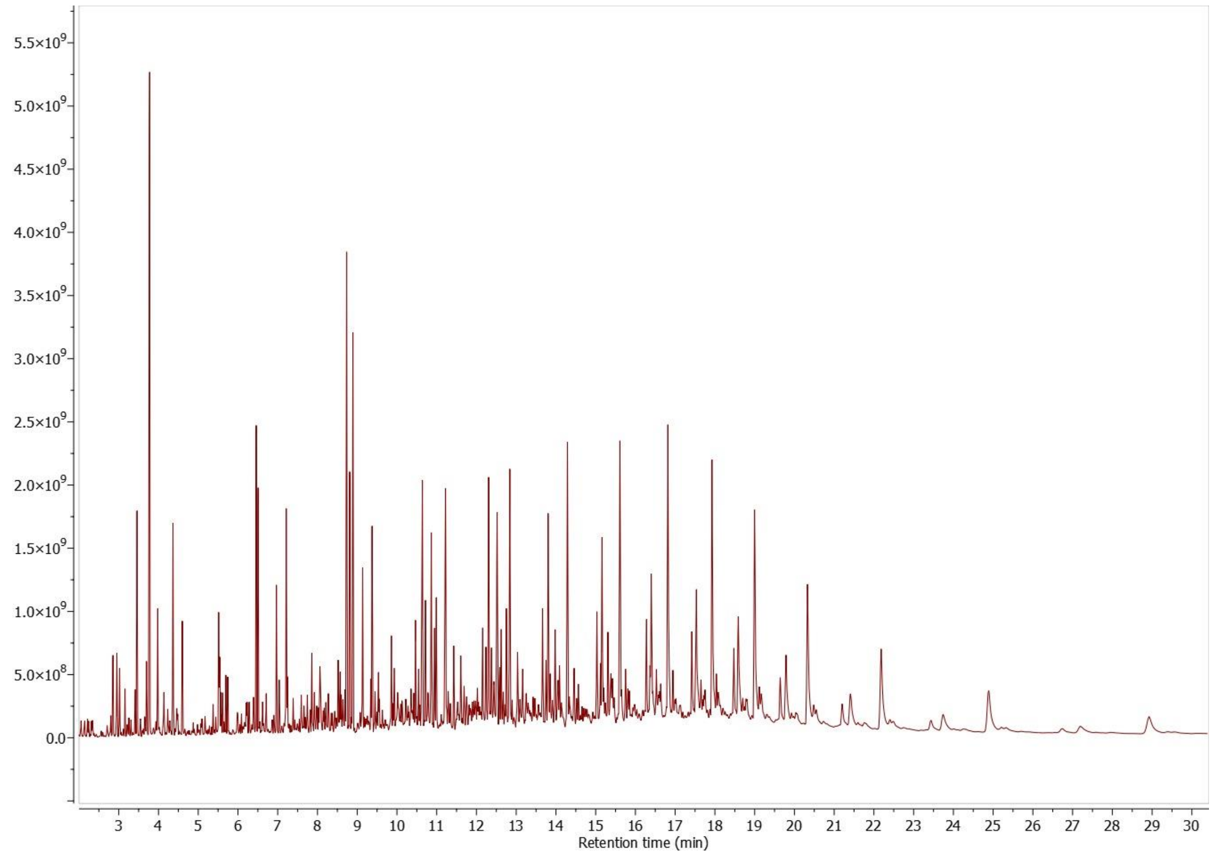

Figure S13: PP liquid pyrolysis product GC-MS data

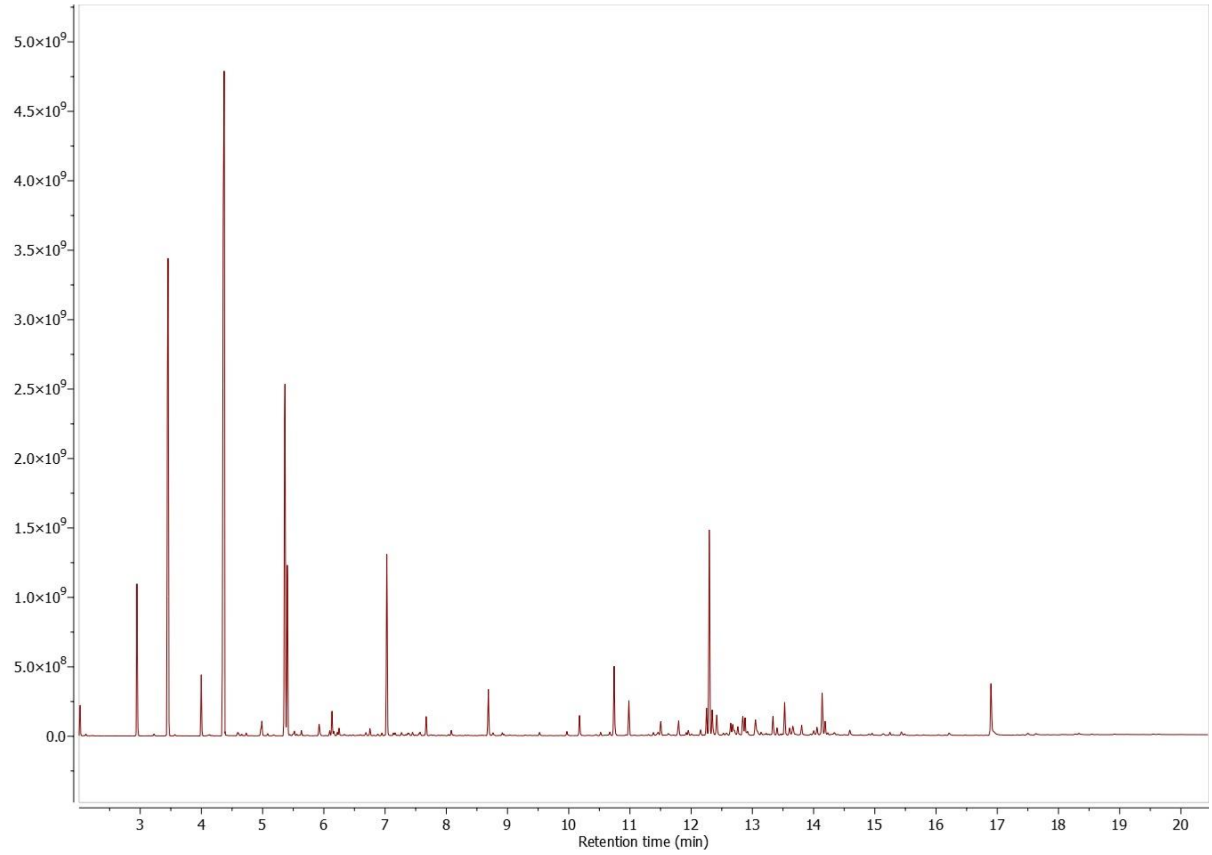

Figure S14: PS:silicone rubber 1:2 liquid pyrolysis product GC-MS data

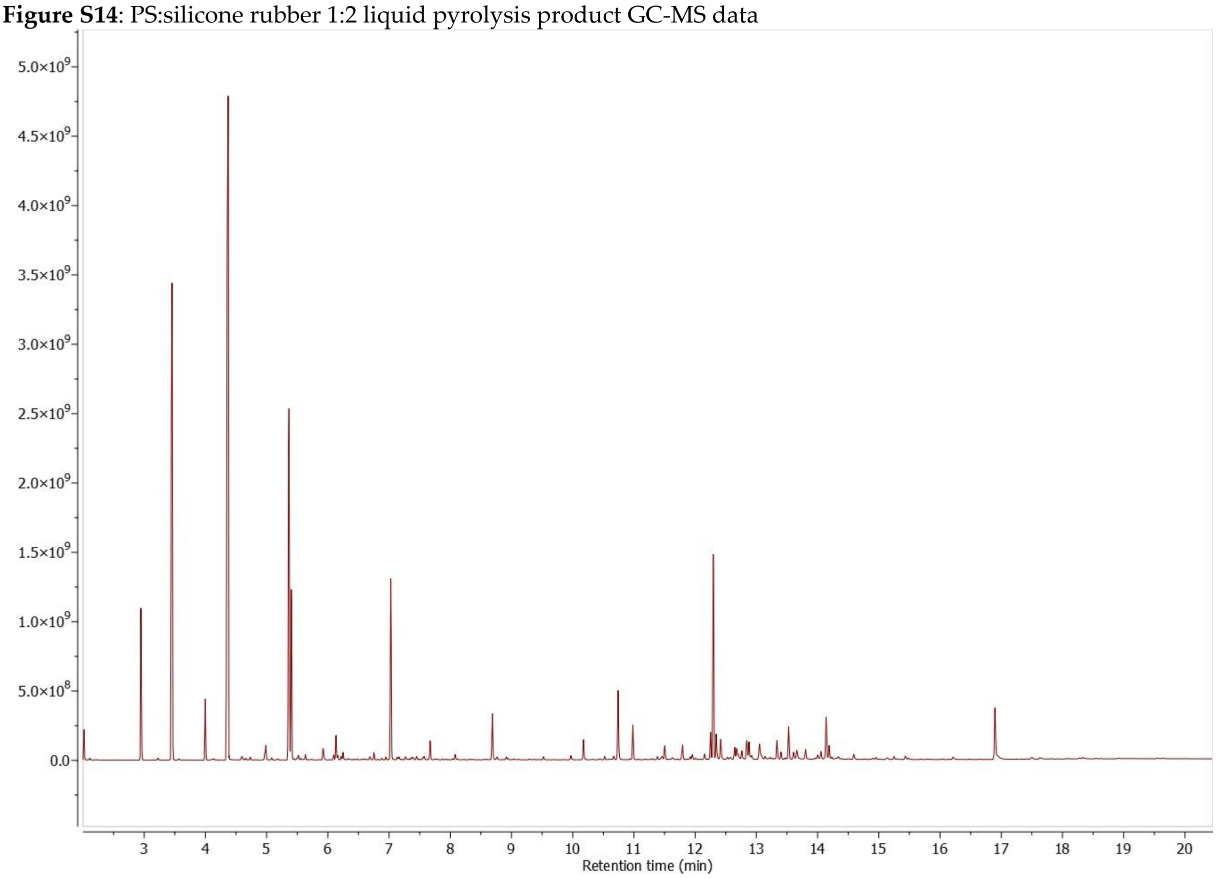

Figure S15: PS:silicone rubber 1:1 liquid pyrolysis product GC-MS data

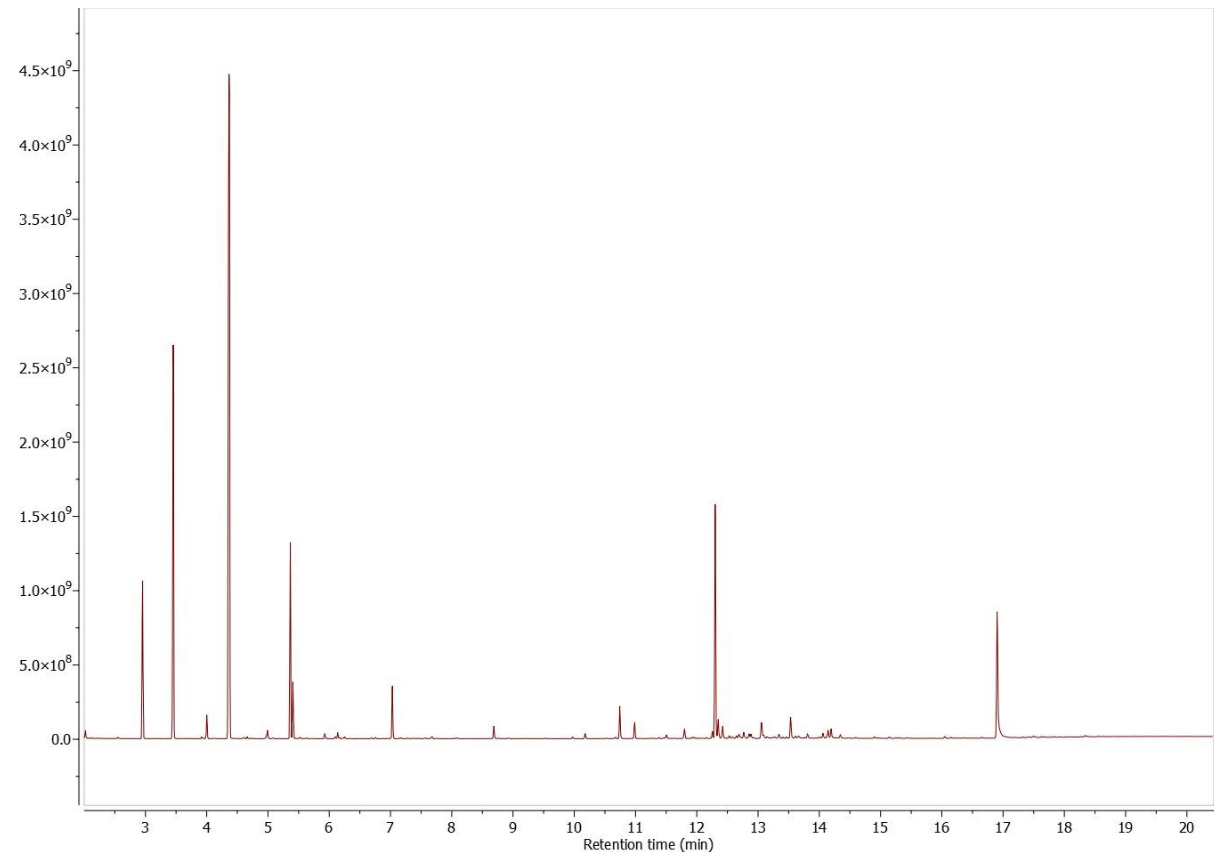

Figure S16: PS:silicone rubber 2:1 liquid pyrolysis product GC-MS data

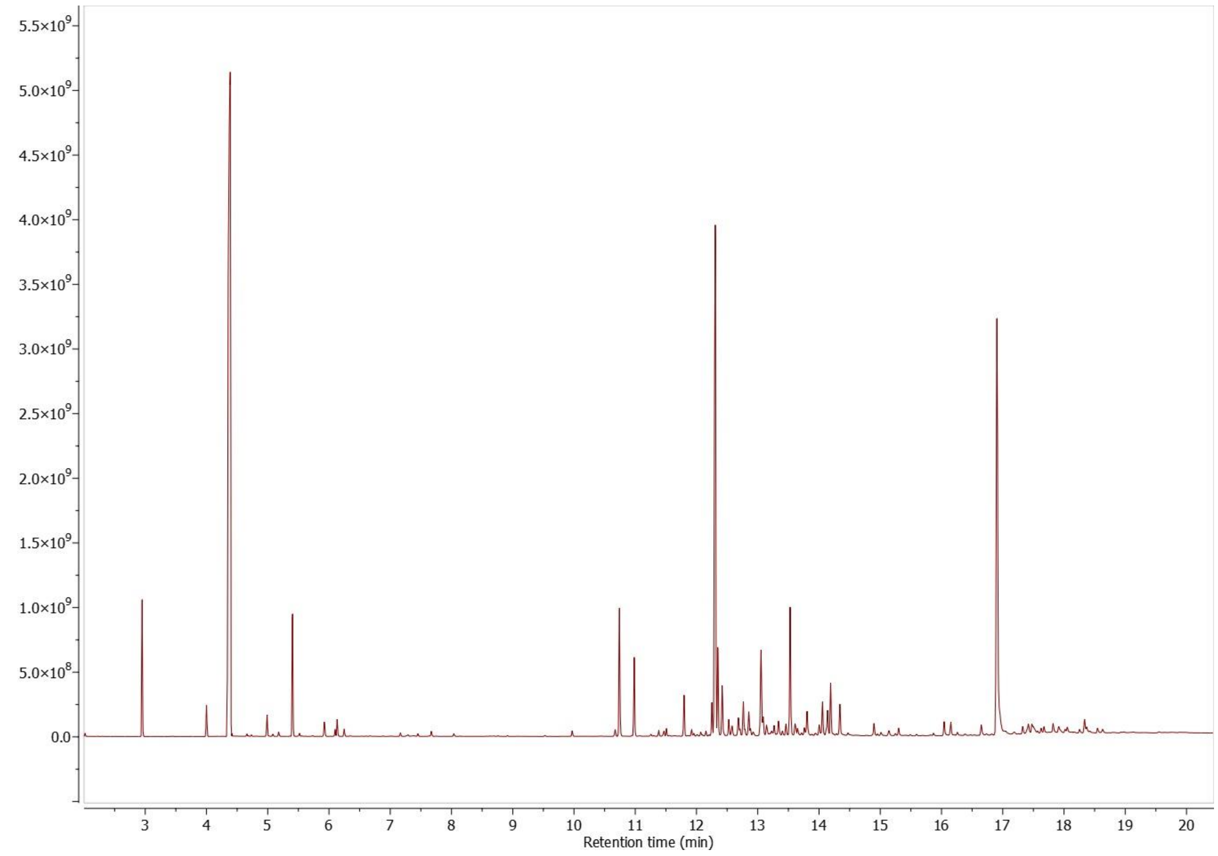

Figure S17: PS liquid pyrolysis product GC-MS data

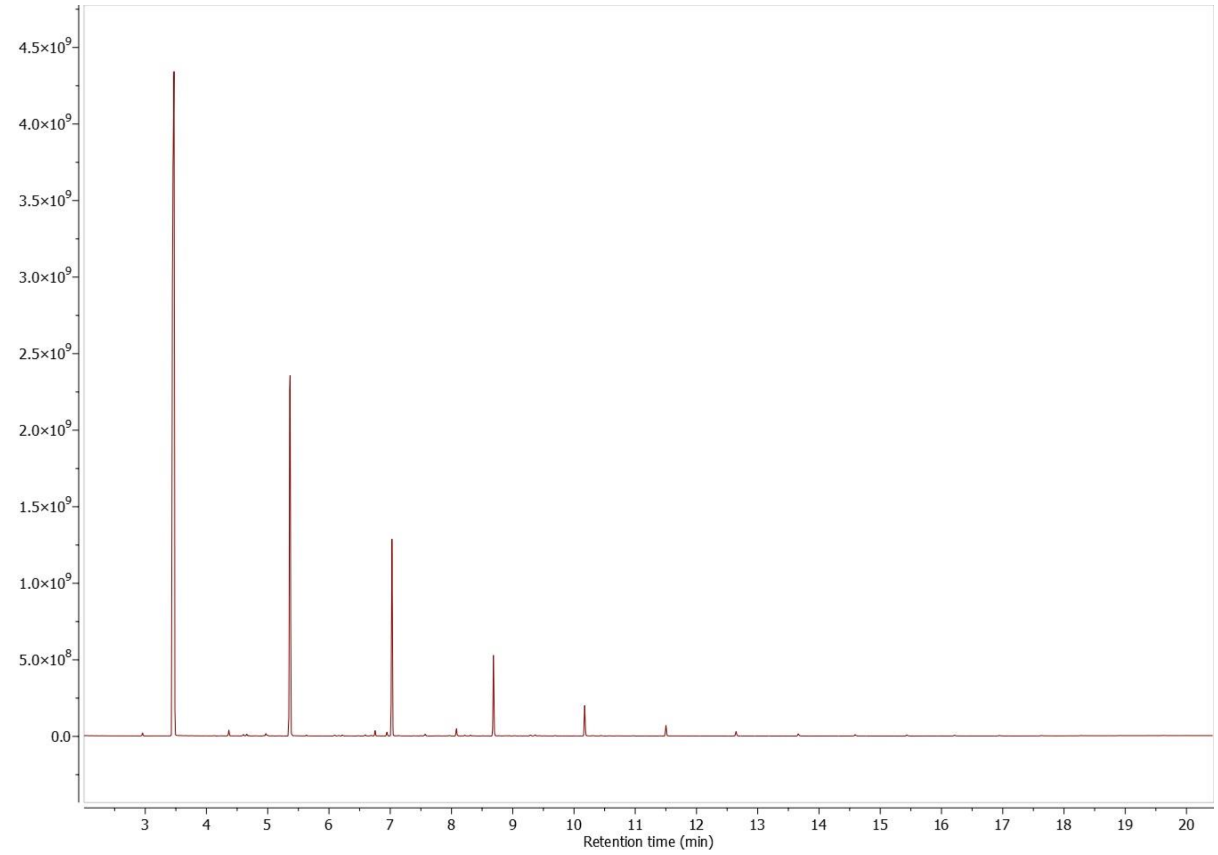

Figure S18: Silicone rubber liquid pyrolysis product GC-MS data

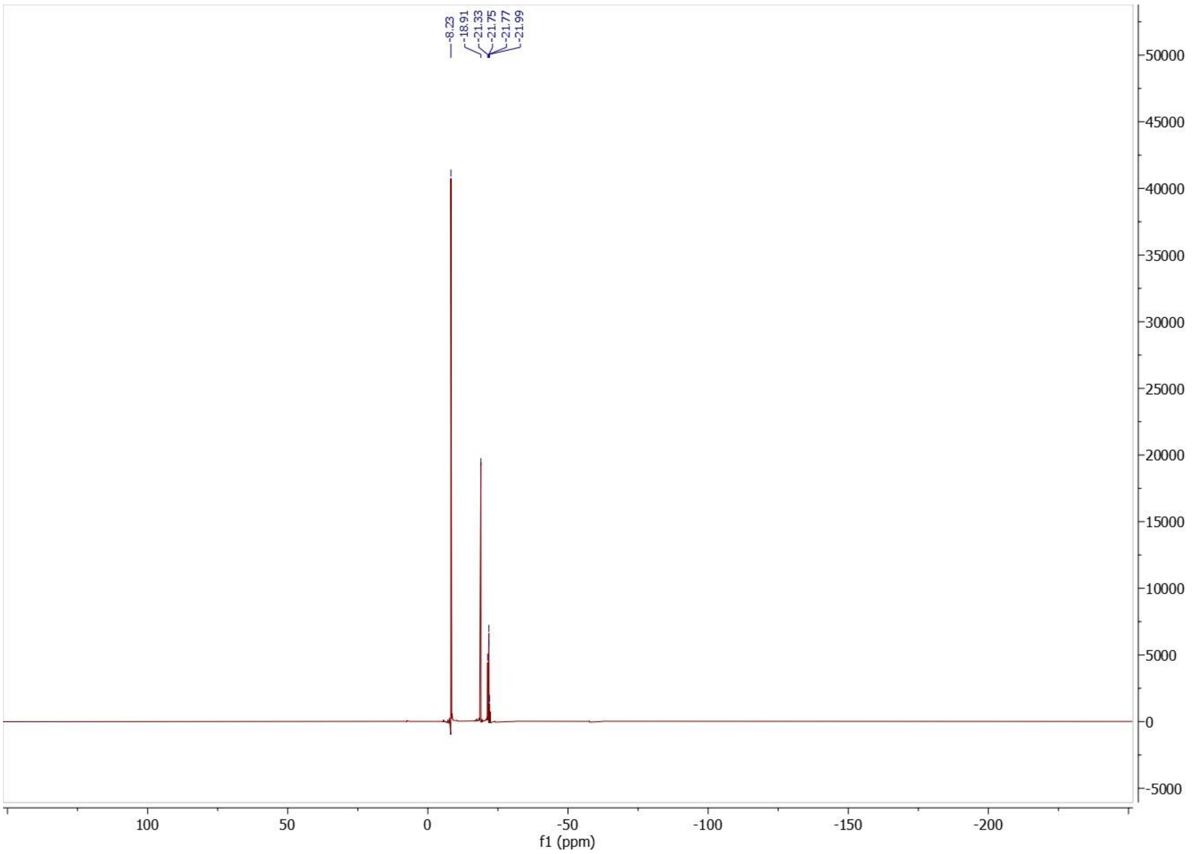

Figure S19: ^{29}Si-NMR spectra of liquid LDPE:Silicone rubber co-pyrolysis product

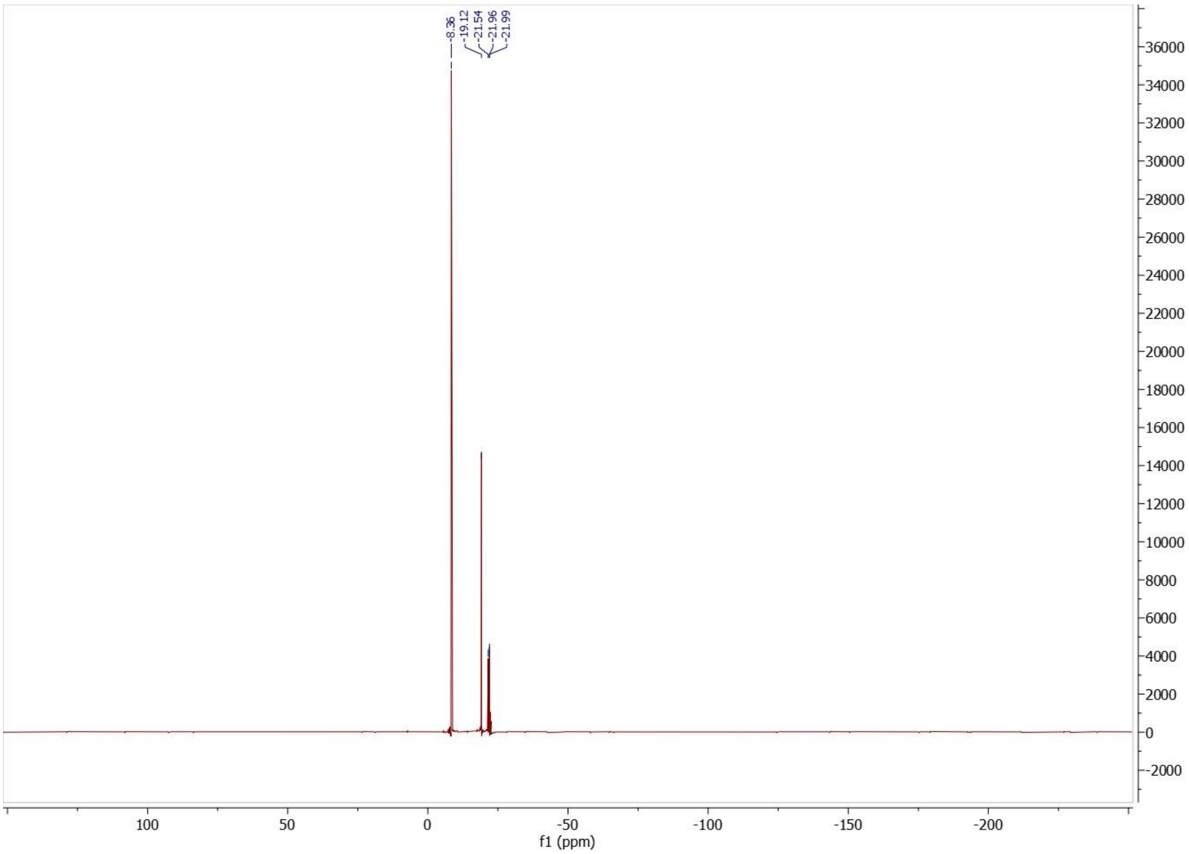

Figure S20: ^{29}Si-NMR spectra of liquid silicone rubber co-pyrolysis product
